# Previously Unidentified Histone H1-Like Protein Is Involved in Cell Division and Ribosome Biosynthesis in Toxoplasma gondii

**DOI:** 10.1128/msphere.00403-22

**Published:** 2022-12-05

**Authors:** Vanessa Severo, Rodolpho Souza, Francisca Vitorino, Julia Cunha, Andréa Ávila, Gustavo Arrizabalaga, Sheila Nardelli

**Affiliations:** a Laboratório de Pesquisa em Apicomplexa, Instituto Carlos Chagas—FIOCRUZ/PR, Curitiba, Paraná, Brazil; b Department of Pharmacology and Toxicology, Indiana University School of Medicine, Indianapolis, Indiana, USA; c Laboratório de Ciclo Celular, Instituto Butantan, São Paulo, Brazil; University at Buffalo

**Keywords:** *Toxoplasma gondii*, chromatin, histone H1, cell division, posttranslational modification

## Abstract

Chromatin dynamics can regulate all DNA-dependent processes. Access to DNA within chromatin is orchestrated mainly by histones and their posttranslational modifications (PTMs). Like other eukaryotes, the apicomplexan parasite Toxoplasma gondii encodes four canonical histones and five histone variants. In contrast, the linker histone (H1) has never been identified in apicomplexan parasites. In other eukaryotes, histone H1 compacts the chromatin by linking the nucleosome and increasing the DNA compaction. H1 is a multifunctional protein and can be involved in different steps of DNA metabolism or associated with protein complexes related to distinct biological processes. We have identified a novel protein in T. gondii (“TgH1-like”) that, although lacking the globular domain of mammalian H1, is remarkably like the H1-like proteins of bacteria and trypanosomatids. Our results demonstrate that TgH1-like is a nuclear protein associated with chromatin and other histones. Curiously, TgH1-like is also in the nucleolus and associated with ribosomal proteins, indicating a versatile function in this parasite. Although knockout of the *tgh1-like* gene does not affect the cell cycle, it causes endopolygeny and asynchronous division. Interestingly, mutation of posttranslationally modified amino acids results in defects in cell division like those in the *Δtgh1-like* mutant, showing that these sites are important for protein function. Furthermore, in the bradyzoite stage, this protein is expressed only in dividing parasites, reinforcing its importance in cell division. Indeed, the absence of TgH1-like decreases compaction of peripheral chromatin, confirming its role in the chromatin modulation in T. gondii.

**IMPORTANCE** Histone H1, or linker histone, is an important protein that binds to the nucleosome, aiding chromatin compaction. Here, we characterize for the first time a linker histone in T. gondii, named TgH1-like. It is a small and basic protein that corresponds only to the C-terminal portion of the human H1 but is similar to histone H1 from trypanosomatids and bacteria. TgH1-like is located in the nucleus, interacts with nucleosome histones, and acts in chromatin structure and cell division. Our findings show for the first time the presence of a histone H1 protein in an apicomplexan parasite and will provide new insights into cell division and chromatin dynamics in T. gondii and related parasites.

## INTRODUCTION

Chromatin is a highly dynamic structure consisting of DNA and proteins, and it is essential for packaging and maintaining genomic DNA. Histone proteins are extremely abundant in eukaryotic cells and, together with nonhistone proteins, modulate its structure, controlling the access to genetic information by DNA-dependent machinery (1, 2). Chromatin is the substrate in epigenetic mechanisms of gene regulation since changes in the interaction between histones and DNA affect chromatin compaction to facilitate or block access of proteins to DNA (1, 3). In eukaryotes, the nucleosome is the basic unit of chromatin and consists of two copies of each of the canonical histones H2A, H2B, H3, and H4, or variants, surrounded by around 147 bp of DNA (4, 5). In addition, the nucleosomes are linked by the histone called H1, or linker histone, stabilizing this structure (6, 7).

Toxoplasma gondii is the etiological agent of toxoplasmosis, a disease that affects up to a fourth of the world’s population (8). During its life cycle, the parasite alternates between felines, the definitive hosts in which it undergoes sexual reproduction, and the intermediate hosts, warm-blooded animals, including humans, in which the parasite undergoes its asexual phase. In intermediate hosts, two stages of the parasite can be found: tachyzoites are responsible for the acute stage, with rapid replication, and bradyzoites, which form tissue cysts, present a low multiplication rate and establish a chronic infection (9). The transition between distinct parasite stages requires precise control of gene expression (10, 11). The tachyzoite-bradyzoite interconversion is coordinated mainly by epigenetic and transcriptional machinery. The main transcription factors of Apicomplexa are ApiAP2, plant transcription factor analogues of the Apetala 2 family, and evidence is emerging that AP2 transcription factors can activate or repress bradyzoite conversion (12–14). On the other hand, alteration of chromatin structure is the most common form of epigenetic regulation. Alteration of chromatin structure changes the interaction of protein complexes with DNA. Chromatin-associated proteins, mainly histone, can undergo posttranslational modifications (PTMs), generating a looser chromatin structure, facilitating the access of transcriptional machinery, or making the chromatin denser, making access to DNA difficult (15). Several canonical factors that are important in chromatin remodeling have been described (3, 14), including the histone acetyltransferases and deacetylases such as *T. gondii* GCN5 (TgGCN5) (16, 17) and TgHDAC3 (18).

Like other eukaryotes, T. gondii possesses all the canonical histones. Histones H3, H4, and H2A are present in a single copy, whereas H2B presents two isoforms—H2Ba, which is expressed mainly in tachyzoites, and H2Bb, which is prominent in sexual stages (19). In addition, five histone variants are present: CenH3, which possesses conserved function as a centromeric histone (20), H3.3, H2A.X, H2A.Z, and a protozoan exclusive variant, H2B.Z (previously known as H2Bv) (21). Along with CenH3, H3.3 and H2A.X are also enriched at the centromere (22). It was reported that H2A.Z and H2B.Z are enriched at transcription start sites, while the H2A.X variant plays a role in gene silencing and DNA repair (23).

Histones are evolutionarily conserved proteins. Nonetheless, histone H1 is the most heterogeneous group of histones, with an evolutionary complexity that reflects distinctive features across species. In all metazoans, histone H1 has a well-conserved central globular domain, a small N-terminal domain, and a long, basic C-terminal domain that adopts a highly folded structure in contact with DNA, affecting the chromatin architecture (24–26). Mammals, for example, have 11 H1 variants, including seven somatic H1 proteins and four germ cell-specific H1 proteins that are regulated during cell development and differentiation (27). The linker histone evolved from eubacteria, but its tripartite structure emerged later in eukaryotes (26, 28). One hypothesis suggests that lysine-rich DNA-binding proteins in eubacteria acquired the globular domain after recombination events (26, 28). This evolutionary complexity means that the structure of H1 can vary in different organisms. For example, H1 from Saccharomyces cerevisiae presents two globular domains, while H1 proteins from dinoflagellates and some parasites, such as kinetoplastids, carry only the C-terminal domain, lacking the globular domain, and are called H1-like (24, 26, 29).

In other organisms, histone H1 is subject to posttranslational modifications (PTMs), which affect the compaction of the chromatin and regulate the expression of specific genes (3). In addition to chromatin-related functions, linker histone has been shown to be a multifunctional protein (24), acting in several cellular processes, such as DNA double-strand break (DSB) repair, when ubiquitylated (30, 31). Moreover, H1 is phosphorylated during cell cycle in several organisms, such as Trypanosoma cruzi and Physarum polycephalum (32, 33). More recently, it has been demonstrated in human and mouse cells that H1 is involved in an extensive network of protein-protein interactions in the nucleolus, with functions related to ribosome biogenesis (34, 35).

Although the repertoire of histones and histone variants in T. gondii genome has been described, histone H1 had not been identified so far. Previous work (36) mentioned a putative histone H1 in T. gondii similar to that of kinetoplastids, but the role of this protein or its function as a linker histone had never been confirmed in T. gondii. Here, we provide experimental evidence that this protein, TgH1-like, functions as histone H1. We demonstrate that the protein is localized in the nucleus with accumulation in the nucleolus and association with chromatin and histones. TgH1-like depletion affects chromatin architecture, decreasing the presence of more tightly packed chromatin at the periphery of the nucleus. Using reverse-genetics analysis, we demonstrate that TgH1-like might be involved in the coordination of parasite replication, since its absence causes asynchronous replication and endopolygeny. In addition, TgH1-like PTM mutants, with mutations that prevent phosphorylation at serine 43 and ubiquitination at lysine 45, showed the same phenotype as the knockout, indicating that PTMs in this protein are important for its function. Furthermore, in bradyzoites, TgH1-like is expressed only in parasites undergoing cell division, reinforcing its role in parasite division.

## RESULTS

### TgH1-like is a nuclear protein associated with chromatin and accumulates in the nucleolus.

**FIG 1:**
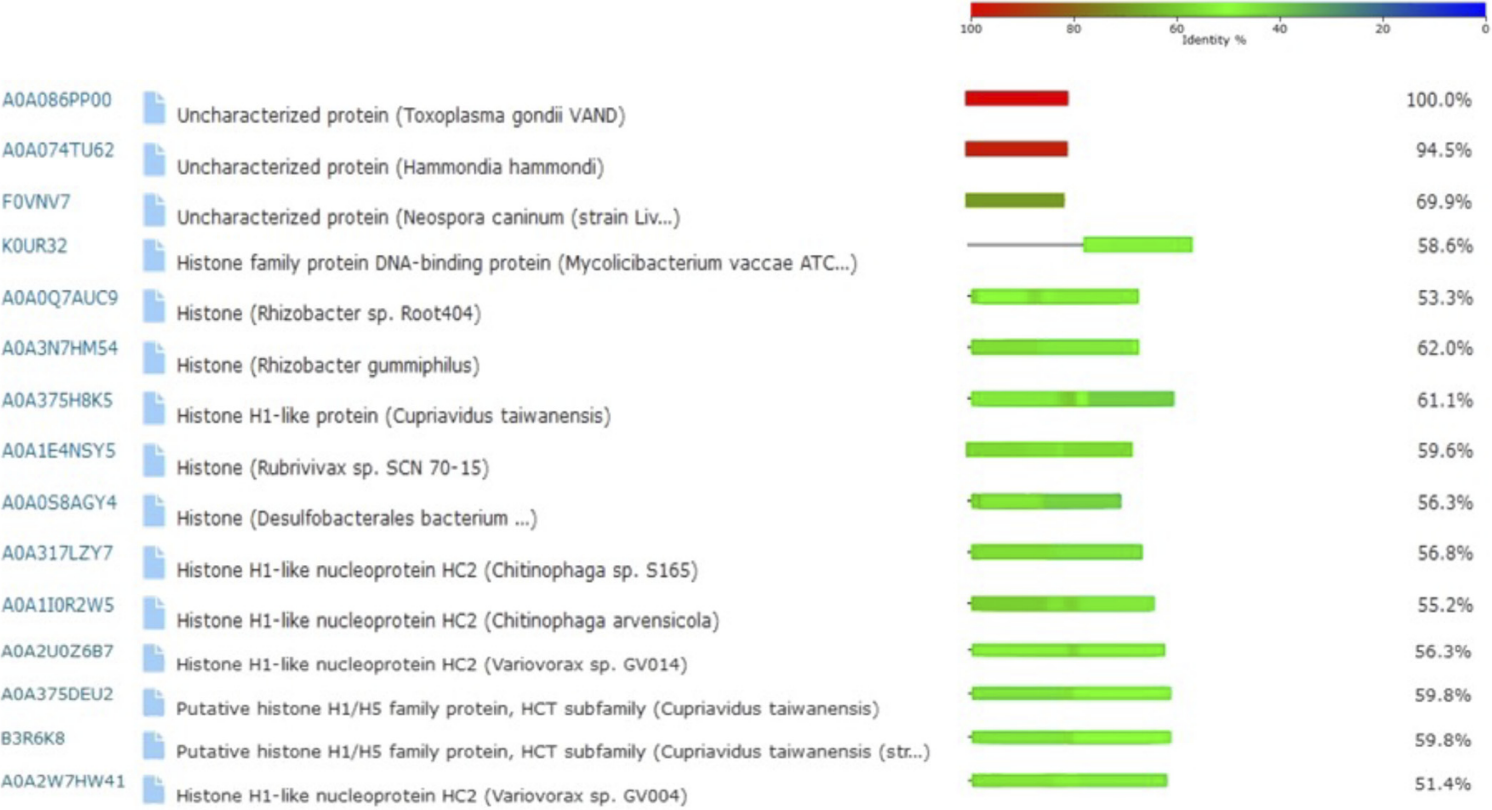
Similarity analysis of TgH1-like by BLAST tool. The sequence of TgH1-like protein (A0A086PP00—uncharacterized protein) was used to search for proteins with sequence similarity using the BLAST tool from UniProt. The color legend indicates the percentage of identity.

**FIG 2:**
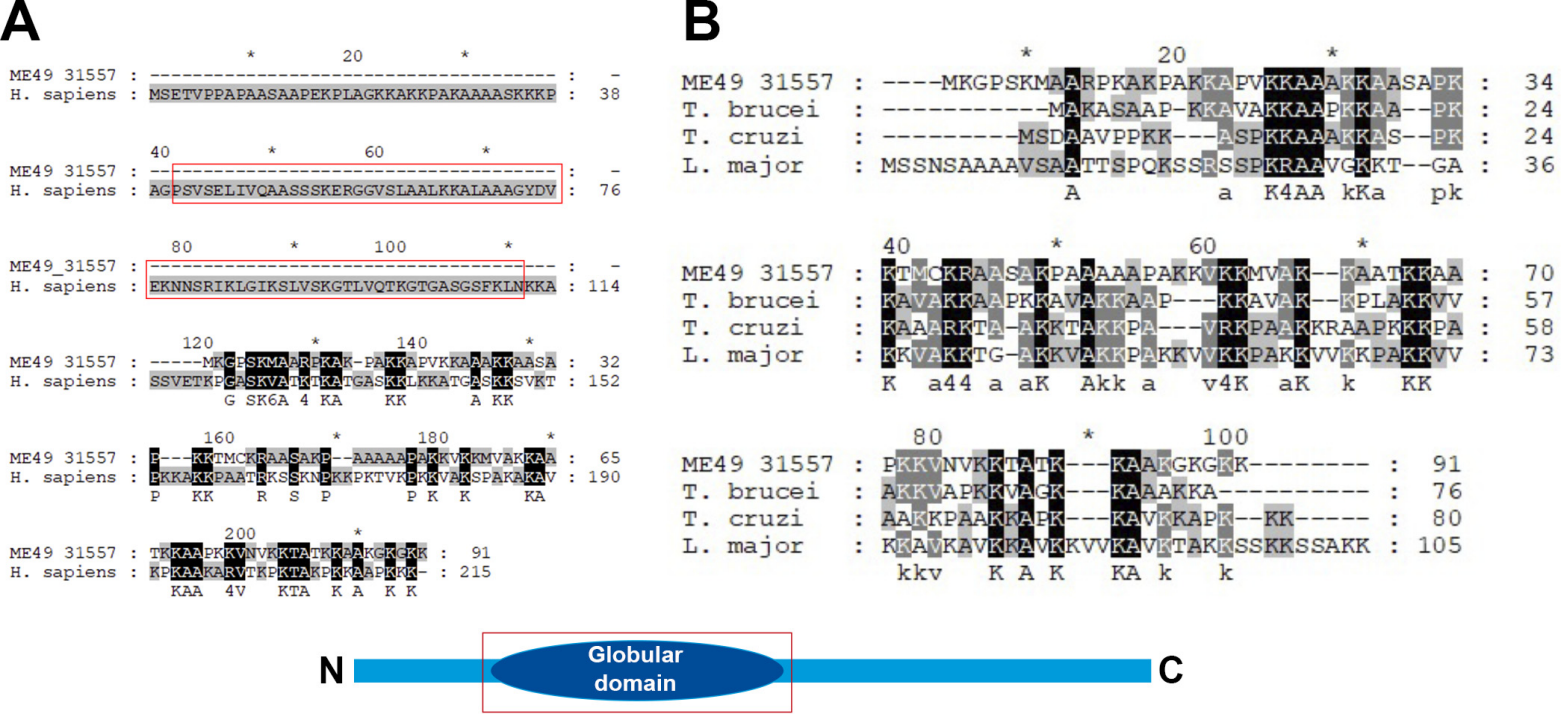
Sequence alignment of TgH1-like with other eukaryotic histones H1. (A) Sequence alignment of TgH1-like (ME49_315570) and histone H1 from H. sapiens (GenBank accession no. AAN06699.1). The red rectangles indicate the region of the globular domain. (B) Multiple-sequence alignment of TgH1-like (ME49_315570) and histone H1-like from kinetoplasts T. brucei (GenBank accession no. CAB76182.1), T. cruzi (GenBank accession no. PBJ81318.1), and L. major (GenBank accession no. CAA11592.1). Below is a schematic of the histone H1 structure from higher eukaryotes.

The sequence of the histone H1 TgH1-like (TGME49_315570) gene is predicted to encode a 91-amino-acid protein (ToxoDB.org) with a higher identity to bacterial H1-like proteins (Fig. 1). Sequence alignment with Clustal Omega also showed 56.76%, 53.25% and 41.86% identities to the H1-like proteins from Trypanosoma brucei, Trypanosoma cruzi and Leishmania major, respectively (Fig. 2). The structure of TgH1-like lacks the globular domain and the N-terminal domain conserved in higher eukaryotes but has similarity to the C-terminal portion of human histone H1 (Fig. 2).

**FIG 3:**
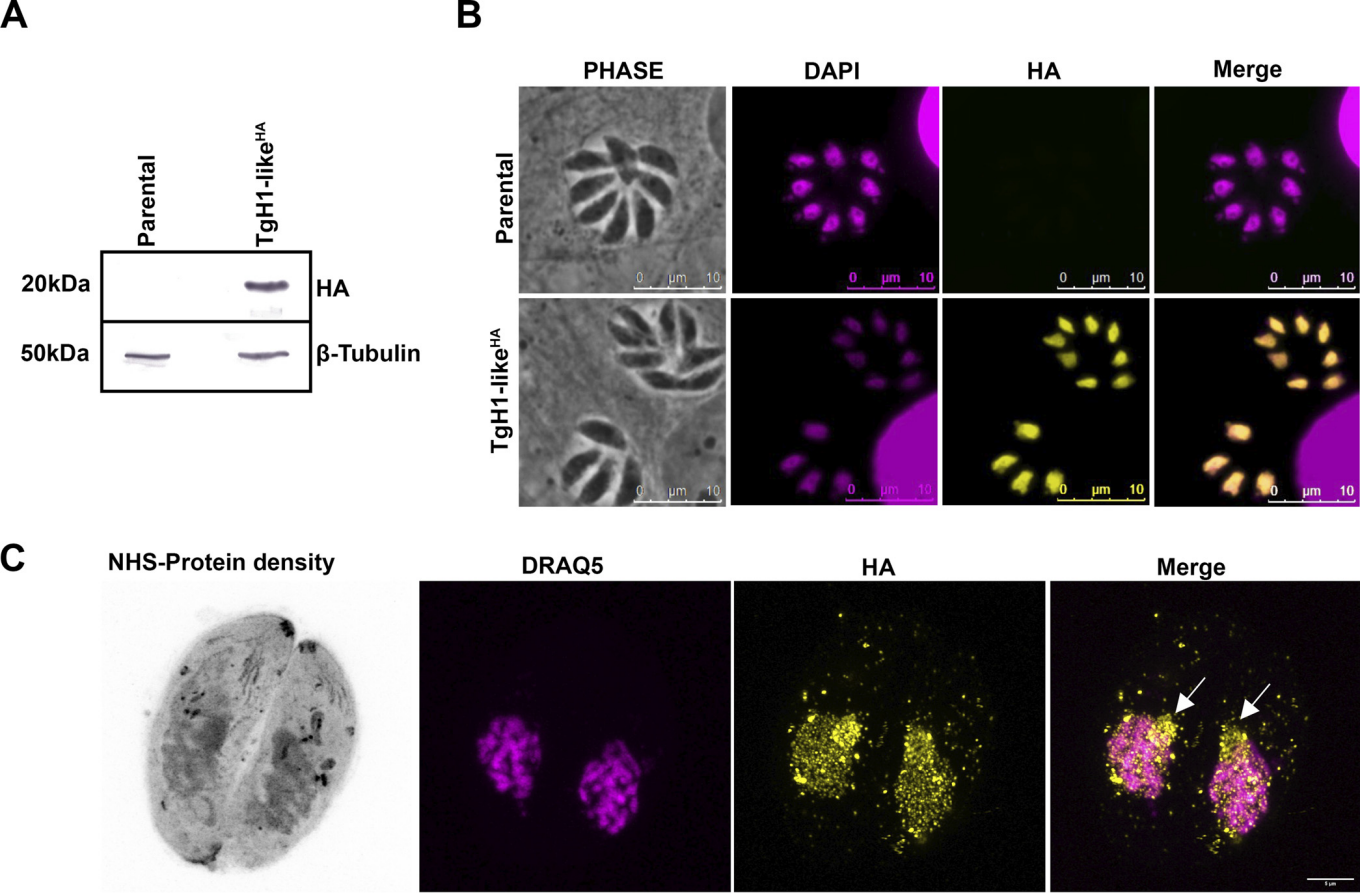
TgH1-like is in the nucleus of T. gondii. (A) Western blot (WB) assay to confirm the expression of HA-tagged TgH1-like (TgH1-like^HA^) corresponding to a 20-kDa protein. The blot was probed with anti-HA and anti-β-tubulin as the loading control. (B) IFAs of parental and tagged parasites stained with anti-HA antibodies (yellow) and DAPI (magenta), which stains DNA in the nuclei and apicoplast. Scale bars = 10 μm. (C) U-ExM of tagged parasites stained with NHS, which detects protein density (grayscale), DRAQ5 to visualize DNA (magenta), and anti-HA antibody (yellow). White arrows indicate the nucleolus. Scale bars = 5 μm.

To determine the localization of TgH1-like, we generated a parasite strain engineered to express a 3-hemagglutinin (3×HA) epitope at the C-terminal end of the endogenous protein (TgH1-like^HA^). PCR confirmed the HA tagging (see Fig. S1 in the supplemental material), and the monoclonal anti-HA antibody recognized a protein of ~20 kDa (Fig. 3A), although the predicted molecular weight is 9 kDa. An unspecific lower band is observed, probably due to overexposure. The hydrophobicity of the protein was verified through the GRAVY (grand average hydrophobicity) score, calculated at https://www.gravy-calculator.de/, where the protein had a low GRAVY score of −0.82, characteristic of hydrophilic proteins. An immunofluorescence assay (IFA) demonstrated that TgH1-like^HA^ has homogeneous distribution throughout the nucleus (Fig. 3B). Using ultrastructural expansion microscopy (U-ExM), it was possible to observe the distribution of TgH1-like^HA^ within the nucleus, showing evidence of accumulation in the nucleolus (Fig. 3C).

10.1128/msphere.00403-22.1 FIG S1 Confirmation of genomic recombination and the presence of the 3×HA tag by PCR. (A) PCR amplification of *tgh1-like* plus HA sequence (1,028 bp) and the HA tag sequence (591 bp). (B) Schematic drawing of homologous integration, indicating the PCR-amplified region for confirmation of HA tag insertion and the expected size. TgH1-like plus HA was amplified using primers Tag TgH1-like - F and HA-Int - R. The HA region was amplified using HA - F and HA - R (see primers in Table S2). The marker was the 1-kb Plus DNA ladder from Invitrogen. Download FIG S1, TIF file, 1.4 MB. Copyright © 2022 Severo et al. 2022 Severo et al. https://creativecommons.org/licenses/by/4.0/ This content is distributed under the terms of the Creative Commons Attribution 4.0 International license.

**FIG 4:**
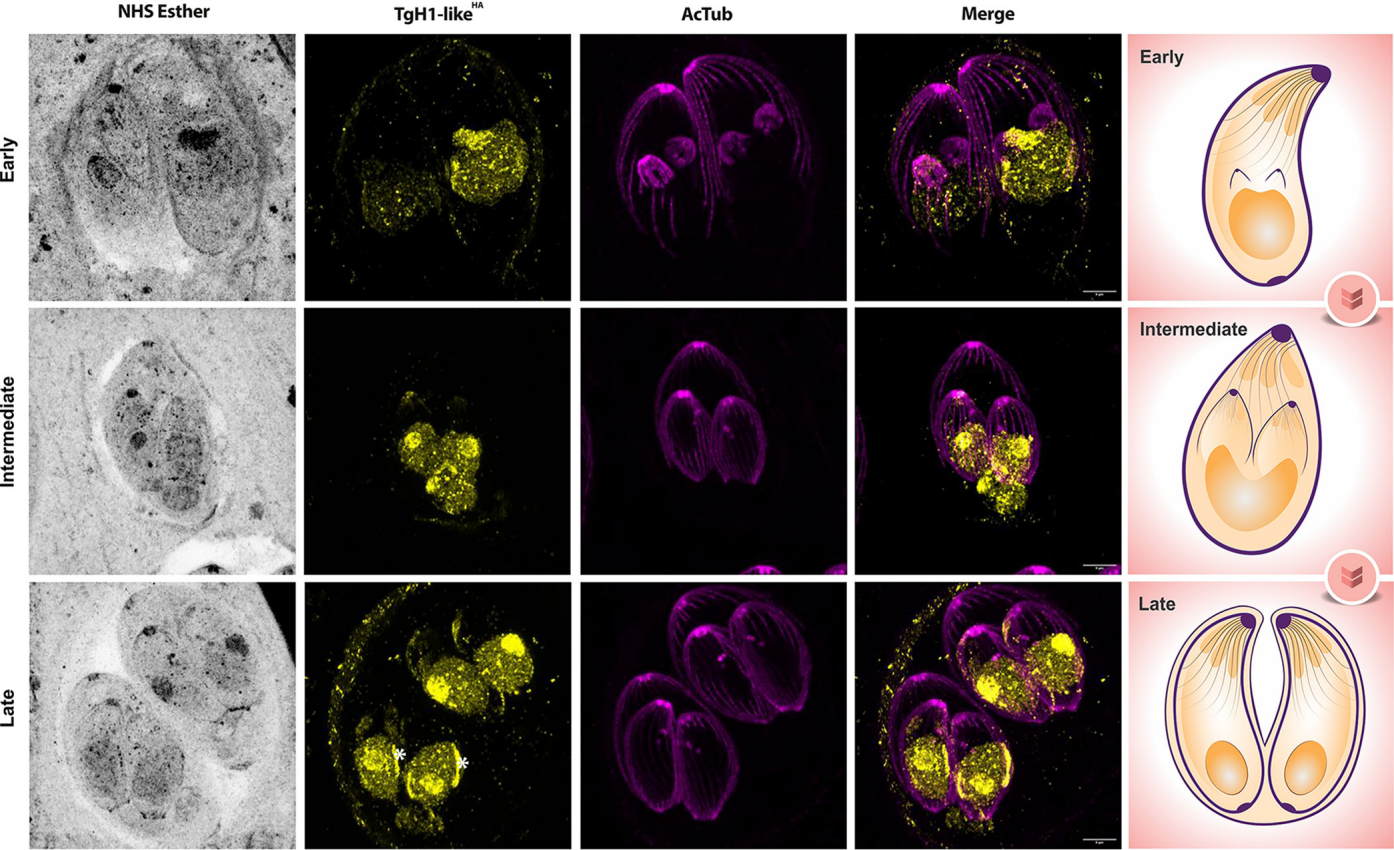
U-ExM of TgH1-like^HA^ in different cell division stages. Tagged TgH1-like^HA^ parasites in early, intermediate, and late cell division stages. Parasites were stained with NHS to detect protein density (grayscale) and antibodies against acetylated tubulin (AcTub) (Lys40) (magenta) and HA (yellow). White asterisks indicate areas of TgH1-like accumulation at the periphery of the nucleus. On the right is a representation of the observed cell division stages. Scale bars = 5 μm.

Ordinarily, T. gondii divides by a process called endodyogeny, where two daughter cells are formed within the mother cell in a coordinated and synchronized manner within the parasitophorous vacuole (37). To determine the distribution of TgH1-like^HA^ during endodyogeny, we monitored its localization at different stages of division using acetylated tubulin, which accumulates in daughter cells, as a marker. During cell division, TgH1-like^HA^ showed the same distribution pattern accumulating in the nucleolus (Fig. 4). Interestingly, at late stages of division we detected accumulation of TgH1-like^HA^ at the nuclear periphery.

**FIG 5:**
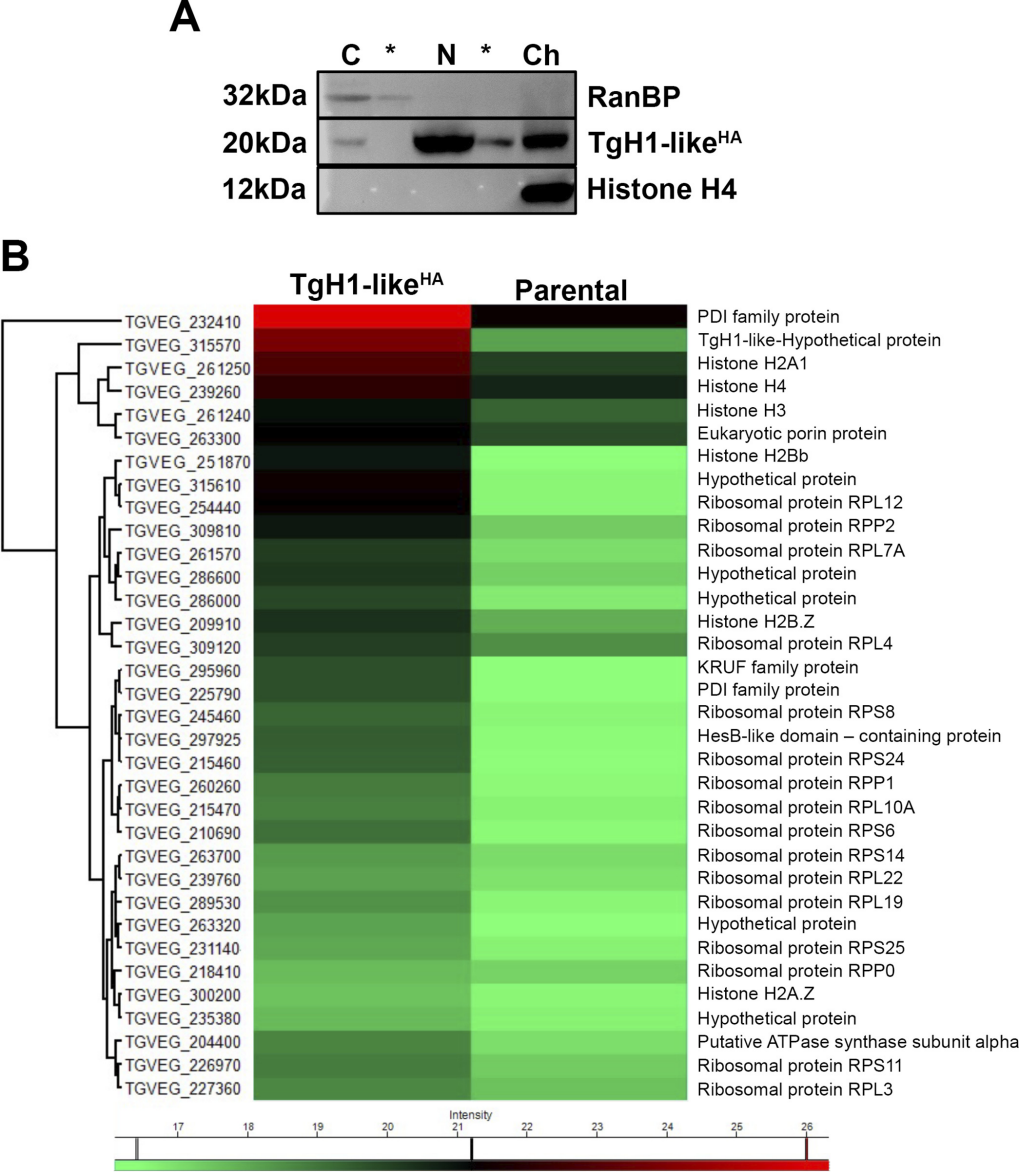
TgH1-like binds to the chromatin along with other histones and ribosomal proteins. (A) A standard acid histone extraction protocol was performed using tachyzoites of the TgH1-like^HA^ strain. Samples obtained from the cytoplasmic (C), nuclear (N), and chromatin (Ch) fractions as well as washing steps (*) were separated in a 15% SDS-PAGE. Western blotting was performed using an antibody against the RanBP, a cytoplasmic protein, H4 as a nuclear control, and anti-HA that recognized TgH1-like^HA^. (B) To confirm the interaction with other histones, an immunoprecipitation assay was performed using whole-parasite extract obtained by cryomilling. Proteins were immunoprecipitated with anti-HA-conjugated beads used as a bait for TgH1-like^HA^ and its complexes. The heat map indicates proteins identified by liquid chromatography-tandem mass spectrometry (LC-MS/MS), with relative abundance represented by color as indicated in the legend (green, lower abundance; red, higher abundance). Missing values were imputed by normal distribution using Perseus.

To confirm the association of TgH1-like with chromatin, we performed a cell fractionation experiment followed by acid extraction, which allows the isolation of chromatin-associated proteins such as histones. Specific antibodies to detect RanBP and histone H4 were used as controls to confirm the purity of the cytoplasmic and nuclear/chromatin fractions, respectively. Importantly, TgH1-like^HA^ fractionated together with other chromatin proteins, such as histone H4 (Fig. 5A, lane Ch). However, TgH1-like^HA^ was also detected in the soluble nuclear fraction (Fig. 5A, lane N), indicating a nuclear function beyond nucleosome binding.

To further confirm the interaction with chromatin proteins, we immunoprecipitated TgH1-like^HA^ using anti-HA antibodies and analyzed the putative partners by mass spectrometry (MS). We also performed the same immunoprecipitation using parental strain parasites as a control. The data were filtered to list only proteins present in at least two of the three samples tested and those with more than 2-fold the normalized intensity values (LFQ intensity values). For the heat map, Z-score values were used, the cytoskeletal proteins were removed, along with those whose ratio with the control was less than 0 (that is, more abundant in the control), which left only those whose ratio was greater than 1 (in relation to the control). A table with protein IDs and fold change values is provided in the supplemental material (Table S1). The list of abundant proteins identified in the H1 complex contains four out of the five canonical histones (H3, H4, H2A1, and H2Bb) and two histone variants H2A.Z and H2B.Z, in addition to several ribosomal proteins (Fig. 5B). Thus, these results strongly suggest an interaction of TgH1-like with the core of histones in the nucleosome and nucleolar proteins.

10.1128/msphere.00403-22.3 TABLE S1 Mass spectrometry proteomics data. Download Table S1, XLSX file, 0.1 MB. Copyright © 2022 Severo et al. 2022 Severo et al. https://creativecommons.org/licenses/by/4.0/ This content is distributed under the terms of the Creative Commons Attribution 4.0 International license.

### TgH1-like knockout leads to asynchronous replication in tachyzoites and changes in chromatin architecture.

**FIG 6:**
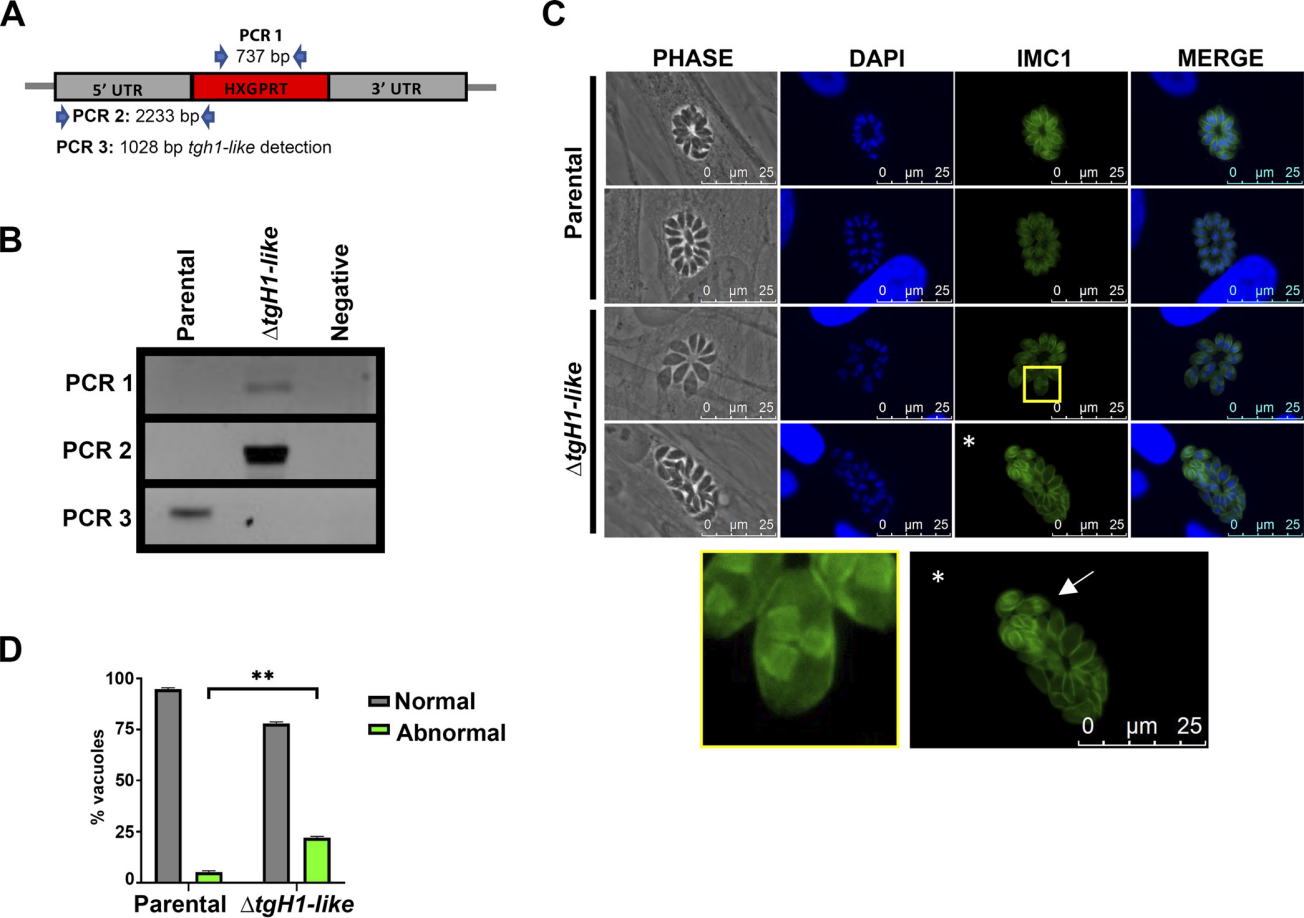
knockout of TgH1-like results in asynchronous replication and endopolygeny. (A) *tgh1-like* knockout parasites were obtained through classical knockout. (B) To confirm the knockout, PCR was performed to detect the selectable marker *hxgrpt* gene (PCR 1), the correct cassette insertion (PCR 2), and the absence of *tgh1-like* (PCR 3) in the *Δtgh1-like* mutant. (C) IFAs of parental and *Δtgh1-like* parasites stained with anti-IMC1 (green). Images were merged with the DNA stain DAPI (blue). In *Δtgh1-like* parasites, the images with asterisks were enlarged. The enlarged yellow box shows parasites with evidence of endopolygeny, while the white arrow shows parasites in asynchronous division. In the parental parasites, these events were not observed. Scale bars = 25 μm. (D) Phenotype counting showing statistical difference in normal and abnormal division between control (TgH1-like^HA^) and *Δtgh1-like* parasites. Unpaired *t* test: **, *P* = 0.0015. Error bars represent standard deviation (SD).

To understand the role of TgH1-like in T. gondii, we generated knockout parasites (*Δtgh1-like*) by homologous recombination to analyze the effect caused by protein depletion. The disruption of the *tgH1-like* locus was confirmed by PCR analyses (Fig. 6B). Flow cytometry analysis shows no significant difference in cell cycle progression between the *Δtgh1-like* mutant and the parental strain (Fig. S2). On the other hand, *Δtgh1-like* tachyzoites stained for inner membrane complex 1 (IMC1) revealed parasites with more than two daughter cells, which is indicative of endopolygeny (Fig. 6C). Furthermore, we noted asynchronous division between parasites within the same vacuole (Fig. 6C). Quantification of these phenotypes was performed using IFA of the TgH1-like^HA^ strain as a control. For this purpose, we consider asynchronous division and endopolygeny events as abnormal division and the absence of these events as normal division. One hundred random vacuoles from 2 independent experiments were evaluated. The phenotype count showed a statistical difference where control parasites presented 5.15% of vacuoles undergoing abnormal division, while knockout parasites presented 21.96% (Fig. 6D).

10.1128/msphere.00403-22.2 FIG S2 Cell cycle analysis of the *tgh1-like* knockout. Shown are results of flow cytometry-based DNA content measurements of Δ*tgh1-like* and control parasites. Cells were fixed in ethanol and stained with propidium iodide (PI). The bar graph shows the percentages of each cell cycle phase, showing no significant difference between *Δthg1-like* and control parasites. Error bars show SD. Download FIG S2, TIF file, 0.3 MB. Copyright © 2022 Severo et al. 2022 Severo et al. https://creativecommons.org/licenses/by/4.0/ This content is distributed under the terms of the Creative Commons Attribution 4.0 International license.

**FIG 7:**
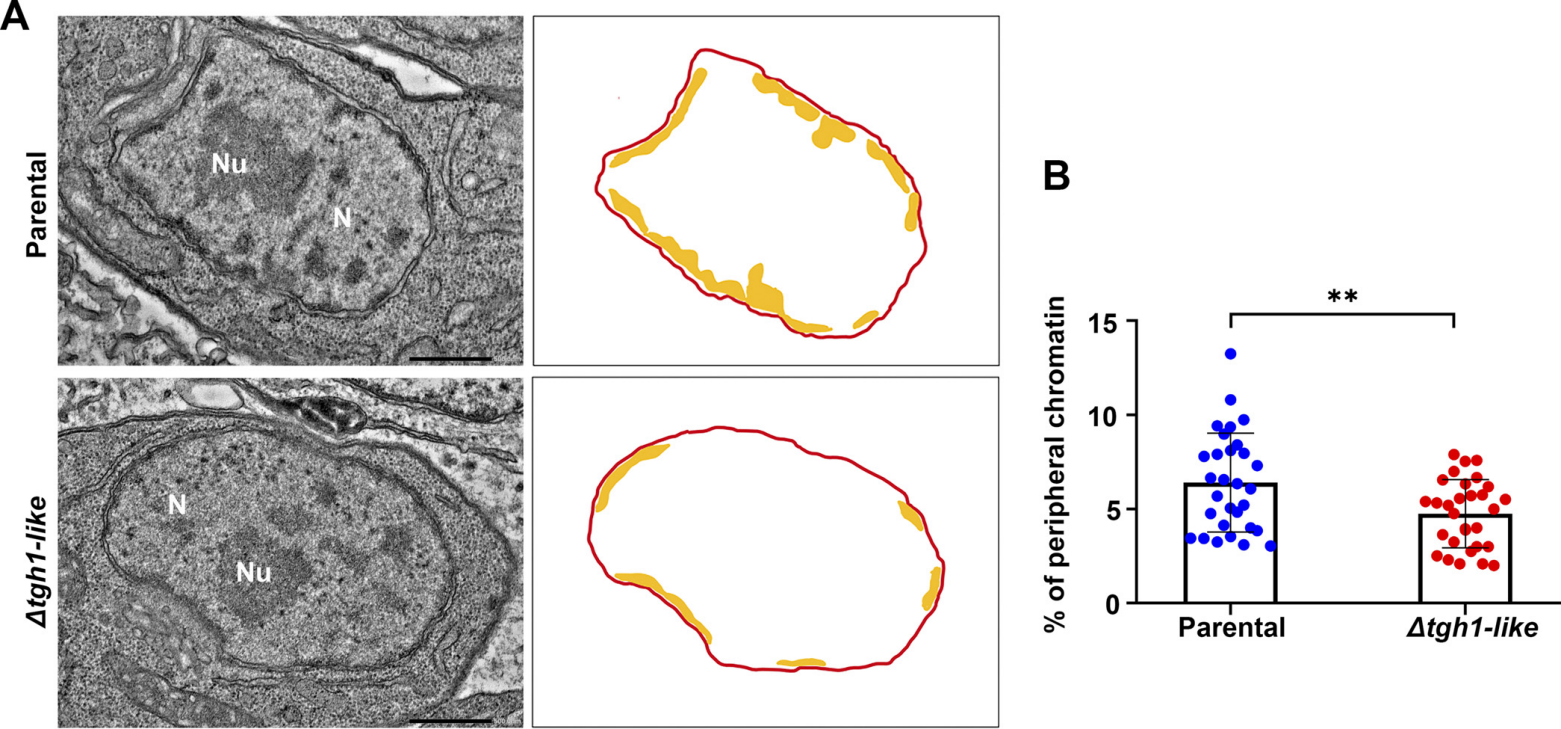
TgH1-like knockout altered the distribution of dense chromatin. Parental and Δ*tgh1-like* parasites were analyzed by transmission electron microscopy (TEM). (A) Images show representative sections of nuclei of parental and Δ*tgh1*-like parasites. The nucleus (N) and nucleoli (Nu) are labeled. On the right is a representation of the quantified areas. The area of the nucleus is shown in red, and the areas of peripheral dense chromatin that were quantified are shown in yellow. Scale bars = 500 nm. (B) The percentage of dense chromatin area at the periphery of the nucleus was quantified using ImageJ software. The Δ*tgh1-like* parasites showed a reduction of dense chromatin on the periphery compared with parental parasites. Unpaired *t* test: **, *P* = 0.0063. Error bars show SD.

One of the main functions of histone H1 is its role in chromatin compaction. Thus, the chromatin of *Δtgh1-like* parasites was analyzed using uranyl acetate *en bloc* stain to highlight nucleic acid (Fig. 7A). In the mutant *Δtgh1-like* parasites, there appears to be a reduction in the proportion of dense chromatin, shown as darker areas, located at the periphery of the nucleus of parasites. To quantify these alterations of chromatin ultrastructure, we collected 30 images of the parental and *Δtgh1-like* strains and analyzed them by the peripheral dense chromatin area (yellow areas in the Fig. 7A representation). For that, we selected the peripheral chromatin using ImageJ software and measured the area that this chromatin occupies in the parental and knockout parasites with reference to the total area of the nucleus. Our results demonstrate that the knockout of TgH1-like caused a significant reduction in dense peripheral chromatin: the parental strain showed 6.40% dense peripheral chromatin, while the knockout showed 4.75% (Fig. 7B). Therefore, TgH1-like appears to have a role in chromatin compaction, especially in peripheral chromatin. In conjunction, our results suggest that TgH1-like may be necessary for correct synchronization of parasite replication in T. gondii and helps in chromatin organization.

### TgH1-like phosphorylation and ubiquitylation sites are essential for synchronized cell division.

**FIG 8:**
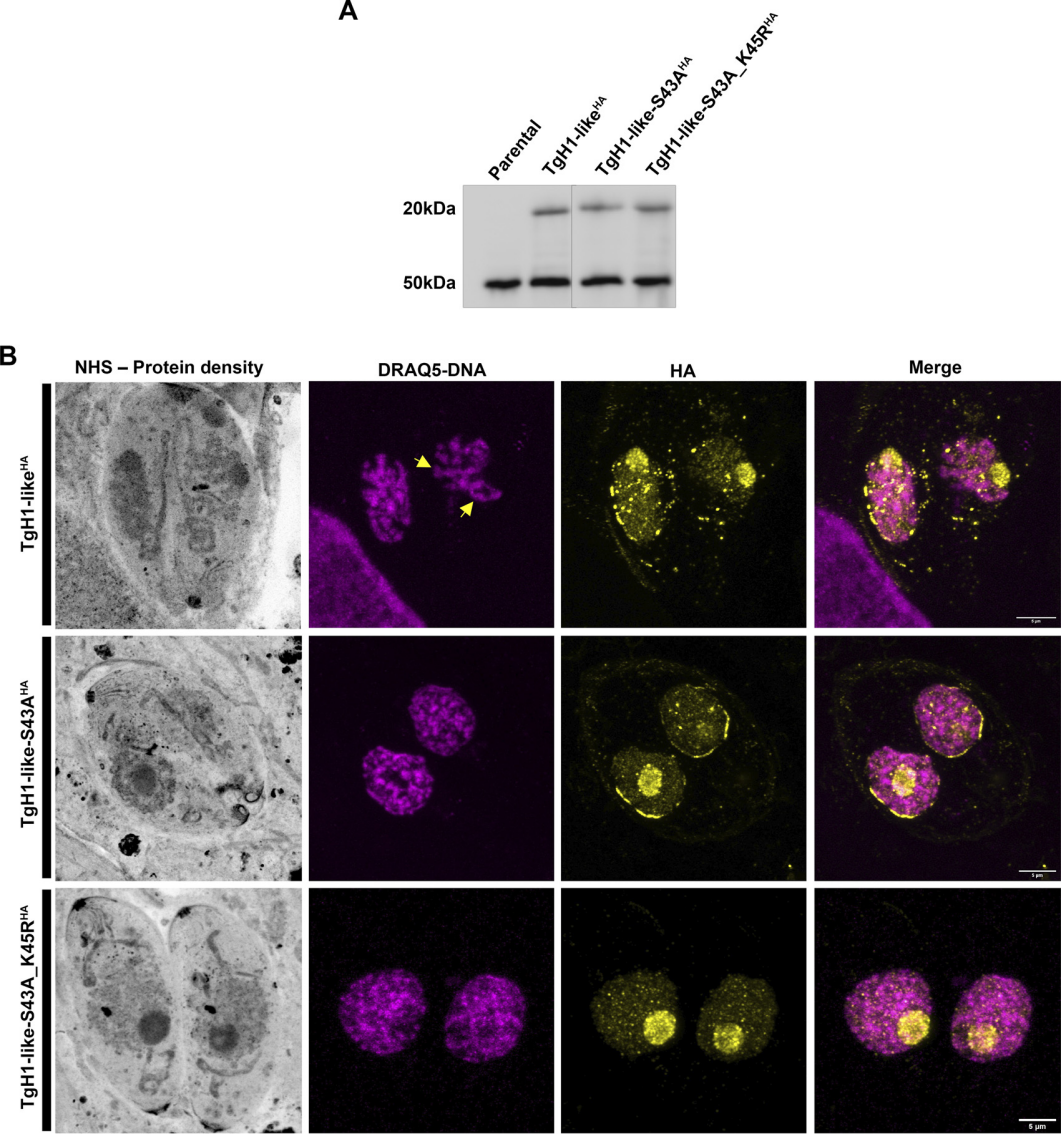
Mutations in PTM sites cause alteration in the protein location and chromatin compaction. (A) Western blot of parental and mutant tachyzoites showing a 20-kDa protein. *T. gondii* β-tubulin was used as a loading control. (B) U-ExM samples were stained for HA (yellow), for DNA with DRAQ5 (magenta), and for protein density with NHS (grayscale). Yellow arrows indicate well-defined areas of compacted DNA. Scale bars = 5 μm.

Another important characteristic of histones, including H1, is their regulation through PTM. According to empirical data present in the ToxoDB database, TgH1-like is subject to ubiquitylation at lysine 45 (38) and phosphorylation at serine 43 (39). To investigate the role of PTMs in the function of TgH1-like, we analyzed the effect of mutating the phosphorylation and ubiquitylation sites. For that purpose, we replaced the endogenous locus by a copy of HA-tagged TgH1-like in which the phosphosite serine 43 is mutated to alanine (TgH1-like-S43A^HA^) or in which serine 43 is mutated to alanine and the ubiquitylated site lysine 45 was replaced by arginine (TgH1-like-S43A_K45R^HA^). The integration of the mutated alleles was confirmed by sequencing. Western blot analysis shows that the mutations did not affect either protein size or expression levels (Fig. 8A).

By U-ExM, we observed that the mutations didn’t cause changes in either the nuclear location or nucleolar accumulation of the protein. On the other hand, the nuclear periphery accumulation of the protein is not observed in TgH1-like-S43A_K45R^HA^ parasites and they seem to have looser chromatin than TgH1-like^HA^ parasites, where the DNA content (DRAQ5 stain) is shown to be more compacted with well-defined areas of DNA, while in TgH1-like-S43A_K45R^HA^ parasites, the DNA content seems to be more spread out (Fig. 8B).

**FIG 9:**
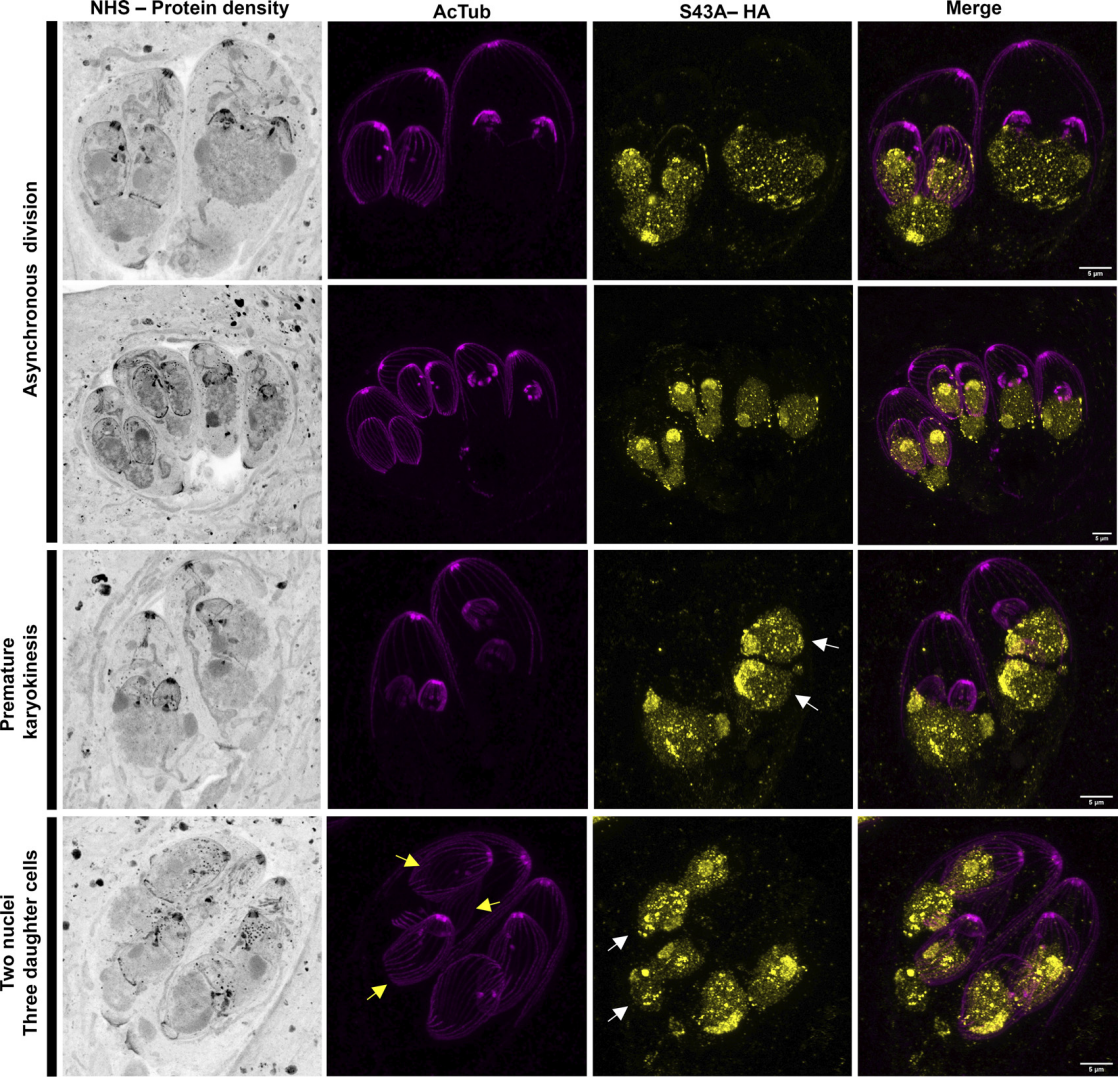
TgH1-like-S43A^HA^ shows a cell division-related phenotype. U-ExM samples were stained for TgH1-like-S43A^HA^ (yellow) and acetylated tubulin (AcTub) (Lys40) (magenta), as well as NHS stain for protein density (grayscale). Images show a representation of cell division-related phenotypes, such as asynchronous division, premature karyokinesis with white arrows indicating two separate nuclei, and cells with two nuclei (white arrows) giving rise to three daughter cells (yellow arrows). Scale bars = 5 μm.

Like the knockout strain, the TgH1-like-S43A^HA^ strain shows asynchronous division between parasites within the same vacuole (Fig. 9). With the TgH1-like-S43A^HA^ strain, it was also possible to observe other phenotypes related to cell division, such as parasites in premature karyokinesis prior to budding of the two daughter cells, when normally the opposite occurs, with daughter cells budding first and karyokinesis occurring near the end of cell division (Fig. 9). Moreover, we observed a mother cell containing two dividing dumbbell-shaped nuclei, with one nucleus giving rise to three daughter cells and the other nucleus giving rise to only one daughter cell (Fig. 9).

**FIG 10:**
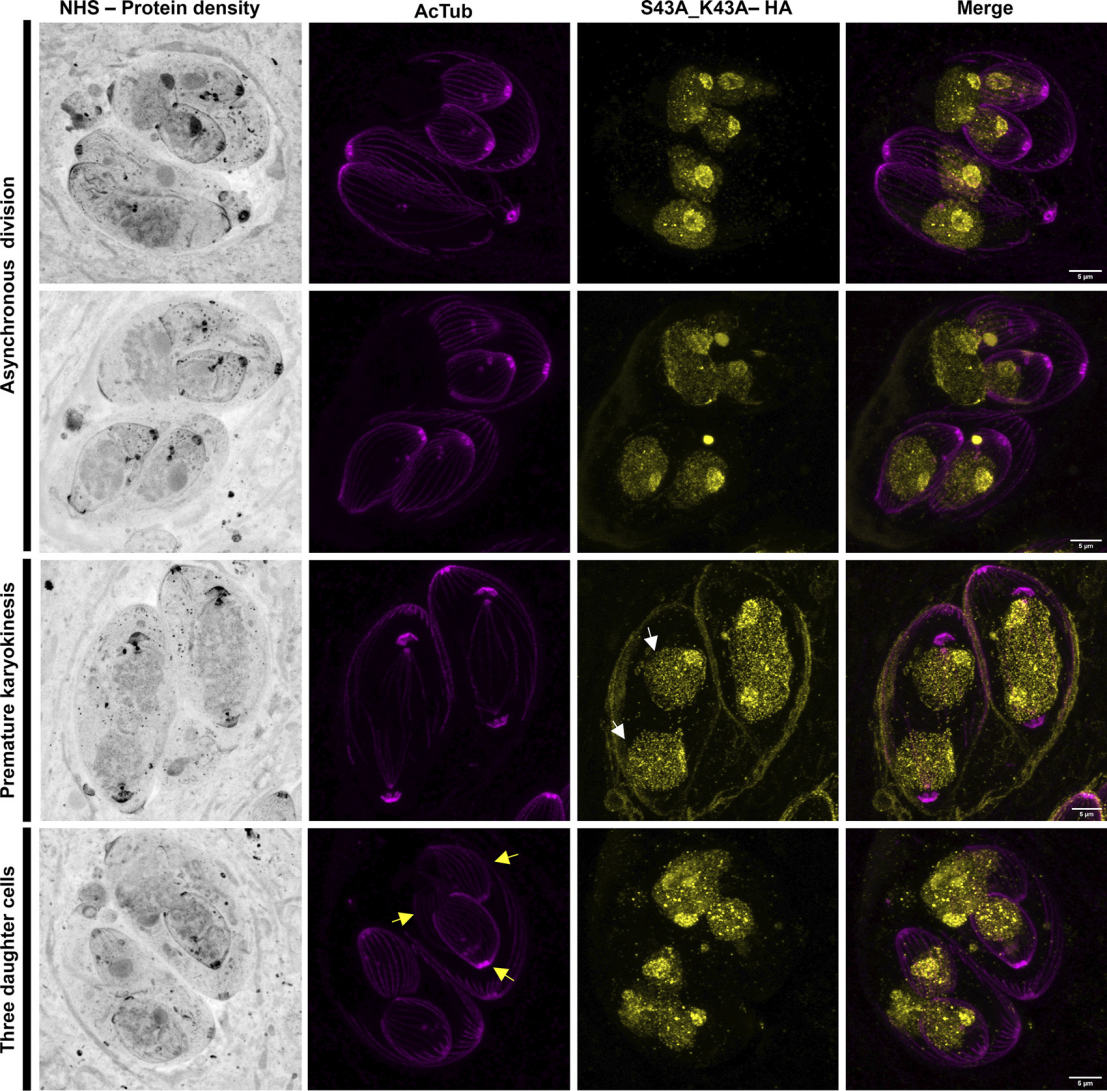
The TgH1-like-S43A_K45R^HA^ strain shows a cell division-related phenotype. Ultrastructure expansion microscopy (U-ExM) samples were stained for TgH1-like-S43A_K45R^HA^ (yellow) and acetylated tubulin (AcTub) (Lys40) (magenta), as well as NHS stain for protein density (grayscale). Images represent phenotypes observed as asynchronous division, premature karyokinesis (white arrows show two nuclei already separate), and endopolygeny events where single mother cells give rise to three daughters cells (yellow arrows). Scale bars = 5 μm.

In the TgH1-like-S43A_K45R^HA^ double mutant, we observe the same phenotypes, including asynchronous division, premature karyokineses, cells with two nuclei, and endopolygeny (Fig. 10).

**FIG 11:**
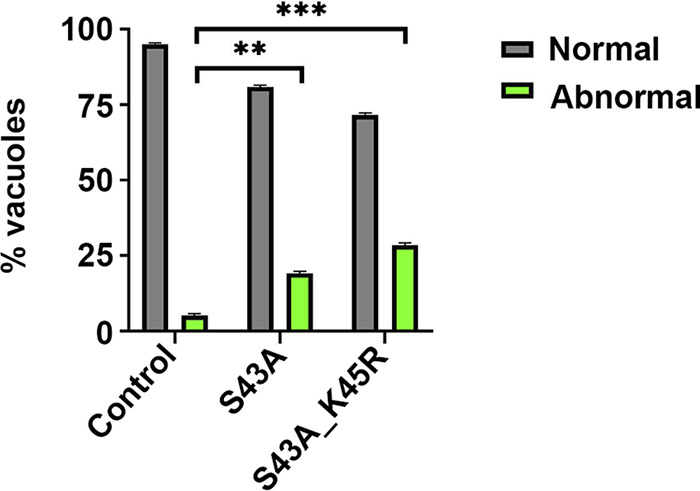
Mutations on TgH1-like PTM sites cause abnormal division. Shown are results of phenotype counting showing statistical difference in normal and abnormal division between the control and TgH1-like-S43A^HA^ parasites (unpaired *t* test: **, *P* = 0.0021) and between the control and TgH1-like-S43A_K45R^HA^ parasites (unpaired *t* test: ***, *P* = 0.0008). Error bars show SD.

Quantification of these phenotypes was performed using IFA of the TgH1-like^HA^ strain as a control and all of the mutant strains. For this purpose, we counted the number of vacuoles showing any of the division-related phenotypes, such as asynchronous division, premature karyokinesis, or endopolygeny, that were considered abnormal division and the absence of any division-related phenotype as normal division within 100 random vacuoles from 2 independent experiments. Control parasites showed 5.15% of vacuoles undergoing abnormal division, while TgH1-like-S43A^HA^ parasites showed 19.15%, and TgH1-like-S43A_K45R^HA^ parasites showed 28.50% (Fig. 11). These results demonstrated that the PTMs on TgH1-like are essential for its function that may be involved in the synchronization and coordination of cell division.

### TgH1-like is present only in bradyzoites undergoing division.

**FIG 12:**
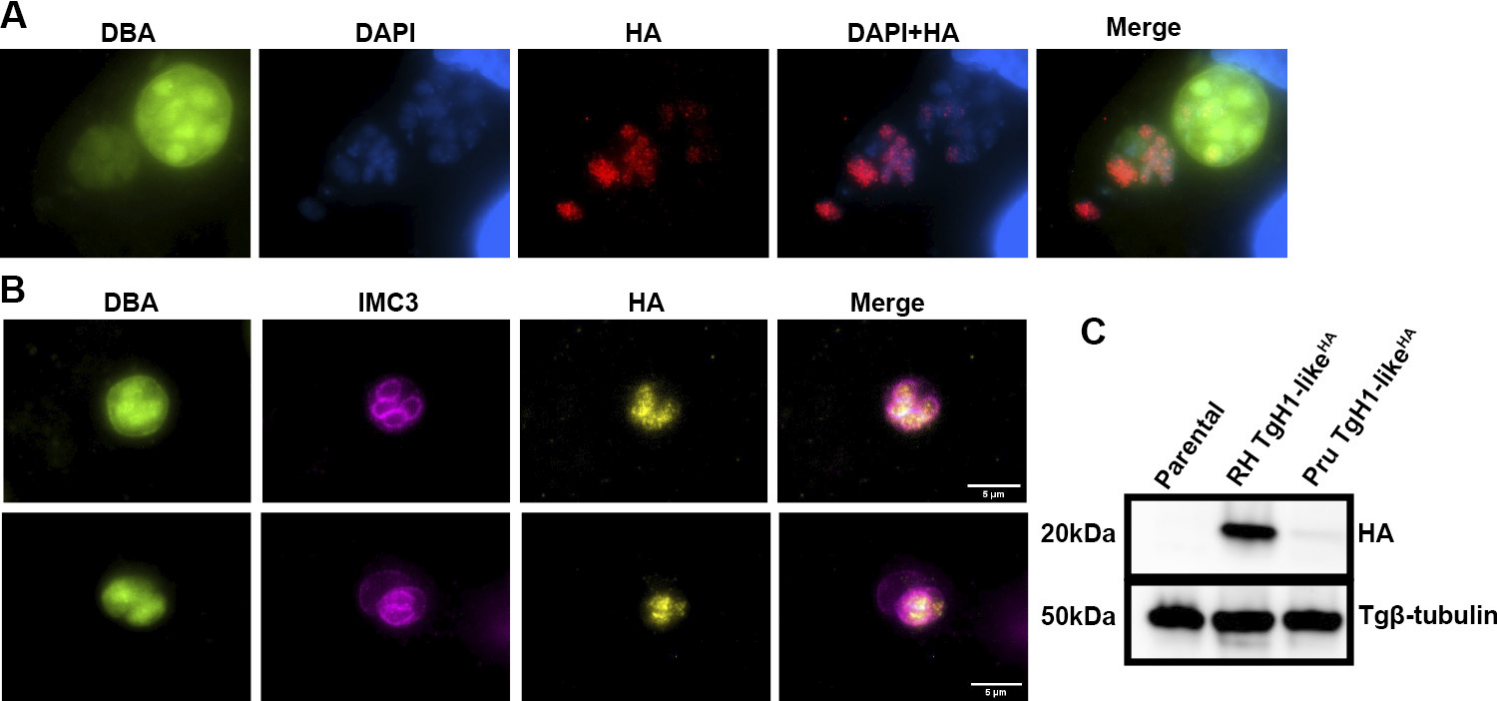
TgH1-like is present only in bradyzoites undergoing division. (A) IFA of Pru TgH1-like^HA^ was performed after 3 to 4 days under bradyzoite conversion. Fluorescein-labeled Dolichos biflorus agglutinin (DBA) was used to visualize cyst walls. (LDH2-GFP reporter is also present in bradyzoites in this channel.) (B) IMC3 antibody was used to observe parasites under division. Scale bars = 5 μm. (C) Western blotting showed a faint band of 20 kDa in Pru TgH1-like^HA^. *T. gondii* β-tubulin was used as a loading control.

While the tachyzoites are the replicative forms with synchronous division, bradyzoites are considered the metabolically and slow-replicating form and are known for their asynchronous replication (40). To verify the dynamic of TgH1-like in bradyzoite, we epitope tagged the endogenous TgH1-like in the Pru Δ*ku80* Δ*hxgprt* strain, which is amenable to producing bradyzoites in tissue culture. IFA of the resulting strain under bradyzoite-inducing conditions was performed using lectin Dolichos biflorus agglutinin (DBA) to detect the bradyzoite cyst wall and IMC3 antibody to detect cell division. The Pru Δ*ku80* Δ*hxgprt* strain used in this work also contains a green fluorescent protein (GFP) reporter in the bradyzoite-specific LHD2 promoter (41). The presence of TgH1-like was not observed in all bradyzoites within the same cyst (Fig. 12A) but is observed in all parasites under division (Fig. 12B). Accordingly, Western blot analysis showed that the expression of the TgH1-like^HA^ was reduced in the Pru strain compared with the RH strain, which can be explained due to the strain-specific phenotype or is likely to the fact that only dividing parasites express the protein in the Pru strain after differentiation (Fig. 12C). These results suggest that TgH1-like has a specific function related to cell division in the bradyzoite form.

## DISCUSSION

Epigenetic control is one of the main mechanisms of gene expression modulation in T. gondii, which is crucial to the differentiation of parasite stages during its life cycle (14, 15). Several canonical factors have been identified as components of chromatin remodeling machinery, mainly histones and histone modifiers (3). However, H1, which functions as a linker of nucleosomes, has never been described in this parasite. Although the structure of histone H1 is not conserved through eukaryote phylogeny, its function has been associated with the modulation of chromatin architecture, resulting in a higher compaction level (27). Here, we describe a protein, named TgH1-like, that is highly similar to the H1 histones of trypanosomes and bacteria. Those histones do not contain the globular domain of human H1 but are involved in chromatin modulation (42).

Microscopy analysis demonstrated that TgH1-like is a nuclear protein, and U-ExM allowed us to observe evident accumulation of TgH1-like^HA^ in the nucleolus and nuclear periphery. Although we observed that HA-tagged TgH1-like migrates as a 20-kDa protein, higher than the predicted sequence of 9 kDa, we confirmed that the HA tag was inserted in the correct region of the gene. Furthermore, the mutation of PTM sites (TgH1-like-S43A^HA^ and TgH1-like-S43A_K45R^HA^) does not alter the protein’s molecular mass in Western blot analysis. There are no other described PTMs in this protein, and thus, it is hard to explain this discrepancy in molecular mass. Maybe this difference can reflect hydrophobicity characteristics, since it is already known that it can alter the protein gel mobility. Shirai et al. have experimentally shown that the hydrophobicity calculated by the GRAVY (grand average hydrophobicity) score (43) influences the protein mobility in gel. This work has described proteins with a different size than predicted when they are analyzed through SDS gel. The hydrophilic proteins (lower GRAVY score) have a lower migration rate, while hydrophobic proteins (higher GRAVY score) tend to migrate faster. In addition, nucleolar proteins, which are generally hydrophilic, have been shown to have a low migration rate (44). Probably, the 20-kDa band observed for TgH1-like can be explained by its physicochemical characteristics, with a low GRAVY score of −0.82, showing features of hydrophilic proteins.

The acid extraction of histones confirmed that TgH1-like is associated with chromatin. However, we observed that TgH1-like is also present in the soluble nuclear fraction, indicating a chromatin-independent nuclear function.

The TgH1-like interaction with histones was confirmed by immunoprecipitation assays followed by mass spectrometry, which identified all of the canonical histones (H3, H4, H2A1, and H2Bb) together with histone variants H2A.Z and H2B.Z in the TgH1-like complex. In T. gondii, histone variant H2B.Z dimerizes with H2A.Z at active genes (23, 45). Altogether, our results demonstrate that TgH1-like shares biochemical and molecular features with histones.

Interestingly, TgH1-like accumulates in the nucleolus and interacts with ribosomal proteins, indicating that it could also be involved in processes related to ribosome biogenesis and cell metabolism. It has been already described that the type of DNA-protein interactions of histone H1 can trigger distinct cellular and biological processes, including the ones related to RNA biogenesis (24, 35, 46). Human H1.0 interacts with an extensive network of proteins that are related to rRNA processing, rRNA assembly, and other biological processes, demonstrating a multifunctional role of linker histones beyond chromatin organization (34, 35, 47). In Drosophila melanogaster, H1 is an essential protein that interacts with ribosomal proteins, such as L22, involved in transcriptional repression (48, 49). Together, these data support the idea that like H1, TgH1-like may be also involved in the rRNA biogenesis.

Heterochromatin generally corresponds to areas of highly condensed chromatin concentrated in the nuclear periphery and around the nucleoli (50, 51). Using U-ExM, we observed that TgH1-like accumulates in periphery regions of the nucleus. In the data obtained by transmission electron microscopy of knockout parasites, in the absence of TgH1-like, there is a reduction in condensed chromatin at the nuclear periphery compared to that in parental parasites. The significant effect observed in Δ*tgh1-like* parasites highlights its function in chromatin organization. Usually, linker histones can change the structure of the chromatin without modulation of gene expression (52, 53). In mammals, for example, H1 depletion altered global chromatin structure when observed by micrococcal nuclease digestion, although microarray analysis showed that the expression of only a few genes was affected (52). In agreement with our results, the knockdown of T. brucei H1 also resulted in a loss of dense chromatin domains (42), reinforcing the role of TgH1-like in the maintenance of structure and organization of chromatin in T. gondii.

H1 histones from several organisms are modulated by PTMs that can be read alone or in combination, generating multiple biological functions (30). TgH1-like has two PTM sites already described with phosphorylation at serine 43 and ubiquitination at lysine 45 (38, 39). Mutating both amino acids altered cell division, resulting in phenotypes like asynchronous division, premature karyokineses, and endopolygeny. In other organisms, such as T. cruzi, H1 is regulated by phosphorylation during the cell cycle. The unphosphorylated form is concentrated in the nucleolus, but when phosphorylated, it starts to spread throughout the nucleus (32). On the other hand, the histone H1 ubiquitylation sites are also associated with DNA damage response. In human cells, it has been shown that ubiquitylated linker histones are recognized by DNA repair factors (31). In T. gondii, the ubiquitinated proteome revealed that ubiquitylated proteins are enriched during the cell cycle, proving an essential regulator of cell cycle progression. The cytoskeletal proteins are a significant fraction of the ubiquitylated proteins, which may be involved in the assembly of the spindle in the late S or M phase, allowing the organization of microtubules. Furthermore, 78% of the ubiquitylated proteins in T. gondii are also phosphorylated, showing a cross talk between the two PTMs (38). It is important to mention that these alterations in cell division in PTM mutants are also observed in TgH1-like knockout parasites. Thus, our data show that phosphorylation at S43 and the combination of phosphorylation at S43 and ubiquitylation at K45 on TgH1-like are essential for its role in cell division, confirming that PTM sites of this protein are important regions for its function.

The association of TgH1-like during cell division was also observed in bradyzoites, where within cysts we could detect TgH1-like mainly in parasites under division. In the tachyzoite, the replicative form, TgH1-like functions in the coordination of cell division, probably due to its role in modulating chromatin structure in addition to being involved in rRNA biogenesis. In bradyzoites, low-replicating parasites show a different control of the expression of this protein.

In apicomplexan parasites, two main modes of asexual division can be defined: internal budding found in cyst-forming coccidia and external budding found in the other parasites (54). We verified the presence of an uncharacterized protein in Hammondia hammondi with 94.5% similarity to TgH1-like, and an uncharacterized protein in Neospora caninum with 69.9% similarity, which may indicate the presence of an H1-like protein in these organisms. T. gondii, *H. hammondi*, and N. caninum perform asexual division in the internal budding mode, comprising endodyogeny and endopolygeny with karyokinesis (54). Thus, the presence of this protein only in coccidia that undergo internal budding cell division mode may be further evidence that it may play a distinct role in that mode of division.

The exact mechanism by which TgH1-like coordinates T. gondii division is still unclear, and further investigation is required. In general, H1 histones are multifunctional proteins involved in different cellular processes (24). The effect observed in this work could be related to different cell maintenance aspects, such as TgH1-like protein-protein interaction aiding in signaling division initiation and coordination sites. As we observed, the absence of TgH1-like proteins or their PTM sites leads to looser chromatin, and the phenotypes also could be related to the protein’s function in DNA-protein interaction, where the absence of TgH1-like could facilitate the expression of genes that are involved in the coordination and timing of cell division.

In conclusion, we have provided evidence that TgH1-like is likely the linker histone in this apicomplexan parasite. Our experimental data demonstrate that TgH1-like has biochemical and molecular characteristics of histones, is present in regions of dense chromatin, and is involved in chromatin organization. The absence of globular domains seems not to affect the interaction of H1 with histones or its role in the chromatin organization. Also, PTM sites seem to be essential for TgH1-like function. Future studies are necessary to understand how TgH1-like modulates cell division. T. gondii has complex epigenetic machinery, suggesting a refined regulation to control gene expression. Modulation in chromatin architecture due to DNA-histone interactions provides the structural basis for epigenetic regulation. The existence of a linker histone in T. gondii brings a new perspective on the regulatory mechanisms of DNA-dependent processes.

## MATERIALS AND METHODS

### Parasite strains and cell culture.

Toxoplasma gondii type I strain RH Δ*ku80* Δ*hxgprt* and type II Pru Δ*ku80* Δ*hxgprt* (LDH2-GFP reporter) were grown in normal human dermal fibroblasts (NHDFs) or human foreskin fibroblasts (HFFs) cultured in Dulbecco’s modified Eagle medium (DMEM) supplemented with 10% fetal bovine serum, 2 mM l-glutamine, 100 U/mL penicillin, and 100 μg/mL streptomycin and maintained at 37°C with 5% CO_2_. To convert parasites to bradyzoites *in vitro*, HFF monolayers were infected with freshly egressed parasites, cultures were maintained in RPMI medium (pH 8.3) in a 37°C incubator with ambient CO_2_, and the medium was replaced daily for 3 to 4 days (55).

### Antibodies and dyes.

The antibodies used in this work and the respective dilutions are as follows: rat anti-HA, 1:500 (Roche); rabbit anti-histone H4, 1:200 (Abcam); mouse anti-RanBP, 1:800 (kindly provided by Hálisson Tesseroli Miot); rabbit anti-β-tubulin, 1:1,000 (Abcam); rabbit anti-IMC1, 1:1,000 (kindly provided by Gary Ward, University of Vermont); rabbit anti-*T. gondii* β-tubulin, 1:5,000 (kindly provided by Michael Reese, University of Texas Southwestern); and rat anti-IMC3, 1:2,000 (56). Alexa Fluor 488-conjugated secondary antibodies (1:2,000; Invitrogen) were used for IFAs. The secondary antibodies used for immunoblot assays conjugated to horseradish peroxidase included the following: rabbit anti-rat IgG, 1:10,000 (Sigma); goat anti-rabbit IgG, 1:10,000 (Sigma); and goat anti-mouse IgG, 1:10,000 (Sigma). Alkaline phosphatase-conjugated antibodies included the following: goat anti-rat IgG, 1:10,000 (Invitrogen); and goat anti-rabbit IgG, 1:10,000 (Invitrogen). For the DNA dye, Dolichos biflorus agglutinin fluorescein-labeled (2 mg at 1:250) (NC1112173 Vector Laboratories) DAPI (4′,6-diamidino-2-phenylindole [5 μg/mL]) was used.

The antibodies and dyes used for U-ExM and the respective dilutions are as follows: rabbit anti-HA, 1:500 (C29F4; Cell Signaling); and mouse anti-acetylated tubulin (Lys40), 1:500; (Sigma-Aldrich). Secondary antibodies and dyes included the following: Alexa Fluor 488-conjugated secondary antibodies, 1:500 (Invitrogen); Alexa Fluor 647-conjugated secondary antibodies, 1:500 (Invitrogen); DNA dye DRAQ5, 1:500 (62251; Thermo Fisher); and Alexa Fluor 405-NHS ester (succinimidyl ester), 1:200 (A30000; ThermoFisher).

### TgH1-like similarity searching.

Genomic sequences of the *tgh1-like* gene (TGME49_315570) were obtained from ToxoDB database (version 28). To identify sequences with similarity to TgH1-like, searches were performed at UniProt (www.uniprot.org/) using the BLAST tool. For sequence alignment analysis, we used the Clustal Omega algorithm (www.ebi.ac.uk/Tools/msa/clustalo/) (57, 58). The following sequences were used for the alignment: Homo sapiens H1 (GenBank accession no. AAN06699.1), Trypanosoma brucei H1 (GenBank accession no. CAB76182.1), Trypanosoma cruzi H1 (GenBank accession no. PBJ81318.1), and Leishmania major H1 (GenBank accession no. CAA11592.1).

### TgH1-like HA tagging and mutations on PTM sites.

The genomic sequence of *tgh1-like* was amplified by PCR from genomic DNA and incorporated into plasmid pLIC-3XHA-HXGPRT via ligation-independent cloning as previously described (59). After plasmid linearization with EcoRV (New England Biolabs), 5 μg of linearized plasmid was precipitated and mixed with freshly lysed 10^7^ tachyzoites of the RH Δ*hxgprt* Δ*ku80* strain previously washed twice in Cytomix buffer (60) complemented with 2 mM ATP. Transfection was performed by electroporation with Nucleofactor II electrode (Amaxa Lonza) in a single pulse using the T16 program in a Gene Pulser cuvette at 0.2 cm in a total of 100 μL (61). After transfection, the parasites were added to confluent NHDF cells for overnight growth. After 24 h, medium was changed to contain 25 μg/mL of mycophenolic acid and 50 μg/mL of xanthine to select for transfected parasites. Parasites were cloned by limiting dilution in 96-well plates, and positive clones were confirmed by Western blotting using anti-HA antibodies as described below. For generation of PTM mutants, the same plasmid used for tagging was edited using the Q5 site-directed mutagenesis kit (NEB BioLabs). The transfection to obtain the PTM mutants was performed by electroporation using the Amaxa P3 primary cell transfection kit in the 4D-Nucleofector (Lonza) in a single pulse using the FI 115 pulse code. All of the primers used in this work are listed in Table S2.

10.1128/msphere.00403-22.4 TABLE S2 Primers used in this study. Download Table S2, DOCX file, 0.01 MB. Copyright © 2022 Severo et al. 2022 Severo et al. https://creativecommons.org/licenses/by/4.0/ This content is distributed under the terms of the Creative Commons Attribution 4.0 International license.

### Immunoblot and immunofluorescence assays.

For Western blot analysis, protein extracts from 10^7^ parasites were prepared in 2× Laemmli sample buffer (Bio-Rad) with 5% 2-mercaptoethanol (Sigma-Aldrich) and loaded in a 15% SDS-PAGE gel to be transferred to nitrocellulose membrane through the Transblot SD semidry transfer system (Bio-Rad). Membranes were blocked in 5% milk–phosphate-buffered saline (PBS)–Tween 20 for 1 h and incubated with primary antibodies (RanPB, histone H4, and HA) for 1 h at room temperature or overnight at 4°C. Membranes were washed three times with PBS-Tween 20 and incubated with secondary antibodies. For antibodies conjugated to horseradish peroxidase, the signal was detected using SuperSignal West Pico chemiluminescent substrate (Thermo Fischer) and captured on an L-pix Chemi Express (Loccus). Alkaline phosphatase-conjugated antibodies were detected with BCIP/NBT (5-bromo-4-chloro-3-indolylphosphate–nitroblue tetrazolium) color development substrate (Promega).

For indirect immunofluorescence, confluent NHDF monolayers grown on coverslips placed in 24-well plates were infected with around 3 to 5 freshly lysed parasites per cell. Cultures were fixed 16 h after infection in 4% paraformaldehyde (PFA) for 20 min, permeabilized with 0.2% Triton X-100 in PBS, blocked for 1 h with 2% bovine serum albumin (BSA) in 0.2% Triton X-100–PBS, and incubated for 1 h at room temperature with primary antibody (anti-HA and anti-IMC). Coverslips were washed in PBS and incubated for 45 min with Alexa Fluor 488-conjugated secondary antibodies (Invitrogen) and DAPI. Samples were mounted using propyl gallate (Sigma) or VECTASHIELD Plus (Vector Laboratories) and visualized using Leica AF6000 with DMI6000 B inverted microscope or a Nikon Eclipse 801 microscope with NIS-Elements AR 3.0.

### Ultrastructural expansion microscopy.

Ultrastructural expansion microscopy (U-ExM) was performed as previously described (62) with a few modifications. Confluent HFF monolayers grown on 12-mm round coverslip 1.5 (NC1129240; Fisher Scientific) placed on 24-well plates were infected with freshly lysed parasites. After 24 h, cells were fixed in 4% PFA–PBS for 20 min at 37°C. Following fixation, the coverslips were washed three times with PBS prewarmed at 37°C and incubated overnight at 37°C in a 1.4% formaldehyde–2% acrylamide (FA/AA) solution in PBS for protein cross-linking prevention. A monomer solution was made on day 1 containing 19% sodium acrylate, 10% acrylamide, and 0.1% *N*,*N*′-methylenebisacrylamide in PBS. The solution was stored as 90-μL aliquots at −20°C. A humidity chamber was prepared with squares of Parafilm and placed in the freezer for 15 min, while aliquots of 10% *N*,*N*,*N*′,*N*′-tetramethylenediamine (TEMED) and 10% ammonium persulfate (APS) were thawed on ice. After, the humidity chamber was transferred onto an ice bucket. The FA/AA solution was removed, and the coverslips were washed with PBS, dried, and placed cell side up onto the Parafilm in the humidity chamber. Five microliters of TEMED and 5 μL of APS were added to 90 μL of monomer solution, the mixture was briefly vortexed, 35 μL of the mixture was added onto the Parafilm for each sample, and the coverslip was quickly flipped with cell side onto the drop and incubated on ice for 5 min followed by 1 h of incubation at 37°C. The gels were transferred to a 6-well plate containing denaturation buffer (200 mM SDS, 200 mM NaCl, 50 mM Tris [pH 9.0]) and incubated for 15 min at room temperature on a platform shaker. The gels were separated from coverslips, placed in Eppendorf tubes filled with denaturation buffer, and denatured at 95°C for 90 min. The gels were transferred to a 10-cm petri dish containing 25 mL of Milli-Q water and incubated for 30 min at room temperature on a platform shaker gently, which was repeated twice for the first expansion round. Then, the gels were shrunk when washed twice with 20 mL of 1× PBS for a 15-min incubation at room temperature on a platform shaker. The gels were then transferred to a new 6-well plate containing 2 mL of blocking solution (3% BSA–PBS) and blocked for 30 min at room temperature on the platform shaker. After blocking, the samples were incubated with 1 mL of primary antibodies in blocking solution overnight at room temperature on the platform shaker. The 35-mm dishes (Cellvis; Fisher catalog no. NC0409658) for imaging were prepared by adding 500 μL of poly-d-lysine (A3890401; Gibco) and incubated for 1 h at 37°C, washed three times with Milli-Q water, completely removed, and stored at 4°C. The gels were washed three times with 2 mL of PBS-T (0.5% Tween 20) for 10 min at room temperature and then incubated with secondary antibodies in PBS for 2 h and 30 min. Next, the gels were washed three times with 2 mL PBS-T for 10 min each, followed by a new round of expansion when the gels were transferred back to the 10-cm petri dishes and washed three times with 25 mL of Milli-Q water for 30 min each. For imaging, small sections of the gel were cut, gently dried, and placed into 35-mm imaging dishes previously coated with poly-d-lysine. The images were acquired on a Zeiss LSM 800 AxioObserver microscope with an Airyscan detector. The Airyscan processing was performed using Zen Blue (version 3.1; Zeiss, Oberkochen, Germany).

### Histone acid extraction.

Histone enrichment was prepared as described by Toro and Galanti (63) with modifications. In brief, 5 × 10^8^ freshly egressed tachyzoites were lysed with buffer A (0.25 M sucrose, 1 mM EDTA, 3 mM CaCl_2_, 0.01 M Tris HCl [pH 7.4], 0.5% saponin, protease inhibitor cocktail [cOmplete tablets, mini EDTA-free, EASYpack; Roche]). The pellet was washed with neutralization/washing buffer B (0.25 M sucrose, 1 mM EDTA, 3 mM CaCl_2_, 0.01 M Tris-HCl [pH 7.4], protease inhibitors). The nuclear-enriched pellet was lysed using buffer C (1% Triton X-100, 0.15 M NaCl, 0.025 M EDTA, 0.01 M Tris-HCl [pH 8.0] in addition to the same protease inhibitors). Proteins weakly bound to chromatin were eliminated by being washed vigorously 3 times with Tris-HCl (pH 8.0). According to the dilutions previously described, Western blot assays were performed using the anti-HA, anti-histone H4, and anti-ranBP antibodies.

### Immunoprecipitation.

For immunoprecipitation assays, the cryogrinding method was used with modifications (64). For this, 10^9^ freshly released parasites were frozen in liquid nitrogen before being added to precooled steel jars containing steel balls to mill the frozen cells into a powder. The resultant cell powder was resuspended in an extraction solution buffer composed of 50 mM Tris-HCl (pH 8.0), 150 mM NaCl, 4 mM EDTA, 1% NP-40, 0.1 mM phenylmethylsulfonyl fluoride (PMSF), and protease inhibitor cocktail (cOmplete Tablets, Mini EDTA-free, EASYpack; Roche). Samples were sonicated in an ultrasonic homogenizer 4710 series sonicator (Cole-Palmer) at the following settings: power 5 for 10 times with 10-s pulses alternating with 1 min on ice. We added 40 μL of anti-HA antibody coupled to magnetic beads (protein A-conjugated Dynabeads; Invitrogen) and maintained it under rotation at 4°C for 3 h to capture the protein. The samples were washed in washing buffer (50 mM Tris-HCl [pH 8.0], 200 mM NaCl, 2 mM EDTA, 1% NP-40, 0.1 mM PMSF, protease inhibitor cocktail [cOmplete tablets, Mini EDTA-free, EASYpack; Roche) and finally eluted with glycine (pH 2.8). The pH of the eluate was adjusted to pH 8.0 by adding 1 M Tris (pH 7.5). The resulting samples were precipitated with 8 volumes of acetone overnight. After being washed with acetone, the samples were dried and analyzed by mass spectrometry.

### Protein digestion and mass spectrometry analysis.

For immunoprecipitation (IP) extracts, dried proteins were digested with trypsin (65). Briefly, samples were resuspended in a mixture of 8 M urea, 75 mM NaCl, and 50 mM Tris (pH 8.2) and digested overnight with sequencing-grade trypsin (Promega) (1:100 [wt/wt] enzyme-protein). Digested samples were cleaned up using C_18_ stage tips. Mass spectrometry analysis was performed as previously described (66). Briefly, peptides were resuspended in 0.1% formic acid and injected in LTQ-Orbitrap Velos (Thermo Scientific) after fractionation on a nano-high-performance liquid chromatography (HPLC) device (NanoLC-1DPlus; Proxeon) equipped with a 5-cm reverse-phase precolumn (5-cm length, inner diameter of 100 μm, filled with a 10-mm C_18_ Jupiter resins; Phenomenex) and a 10-cm reverse-phase capillary emitter column (inner diameter of 75 μm, filled with 5-mm C_18_ Aqua resins; Phenomenex). Peptides were separated with a gradient of 2 to 35% acetonitrile in 0.1% formic acid for 52 min followed by a gradient of 35 to 95% for 5 min at a flow rate of 300 nL/min. The source voltage and the capillary temperature were set at 1.9 kV and 200°C, respectively. The mass spectrometer was operated in a data-dependent acquisition mode to automatically switch between one Orbitrap full scan and 10 ion trap tandem mass spectra. The Fourier transform (FT) scans were acquired from *m/z* 200 to 2,000 with a mass resolution of 30,000. Tandem mass spectrometry (MS/MS) spectra were acquired at a normalized collision energy of 35%. Singly charged and charge-unassigned precursor ions were excluded. The raw data were processed using MaxQuant (67) version 1.6. Proteins were identified by searching against the complete database sequence of T. gondii VEG (DB-43). Carbamidomethylation (C) was set as a fixed modification, while oxidation (M) and acetylation (N terminal) were set as variable modifications: also used were maximal missed cleavages of 2, MS1 tolerance of 20 ppm, MS2 of 0.5 Da, and maximum false peptide and protein discovery rates of 0.01. The “proteingroups.txt” output was analyzed by Perseus (67), and proteins classified as contaminants and/or identified only by modified peptides were filtered out. Additional filtering steps included removal of proteins only identified at control immunoprecipitates (31 proteins), proteins with a log_2_ ratio (H1/control) lower than 1 (32 proteins), and cytoskeleton proteins (5 proteins). Relative protein quantitation was performed using the LFQ algorithm of MaxQuant (68) using a minimum ratio count of 1.

### TgH1-like knockout.

The TgH1-like knockout lineages (*Δtgh1-like*) were generated by the complete replacement of the coding sequence by *HXGPRT* (59). Therefore, primers were designed to amplify around 2 kb of *tgh1-like* flanking regions. The knockout cassette was amplified by PCR and transfected in tachyzoites from the RH Δ*hxgprt* Δ*ku80* strain, and positive clones were selected as described above. The positive clones were confirmed by PCR analysis. Primers were designed to confirm the insertion of *hxgprt.* The absence of the *tgh1-like* gene was confirmed with primers used to amplify the *tgh1-like* gene. The presence of the *hxgprt* selectable marker was confirmed by PCR. All of the primers used in this work are listed in Table S2.

### Transmission electron microscopy.

Cells infected with tachyzoites were fixed in a modified Karnovsky solution (69) containing 2.5% glutaraldehyde, 4% paraformaldehyde, and 0.1 M cacodylate buffer for 16 h at 4°C. After fixation, they were dehydrated through a series of different ethanol concentrations and incubated for 2 h at room temperature in 2% uranyl-acetate protected from light. They were embedded in EMbed 812 resin. Ultrathin sections were examined after staining with lead citrate and uranyl in a JEM-1400Plus electron microscope. Thirty images of each sample were captured, and the percentage of area referring to dense peripheral chromatin was measured using ImageJ software, considering the nucleus area as 100%.

### Data availability.

The mass spectrometry proteomics data have been deposited in the ProteomeXchange Consortium via the PRIDE (70) partner repository under the data set identifier PXD037318.

## References

[1] Khorasanizadeh S. 2004. The nucleosome: from genomic organization to genomic regulation. Cell 116:259–272. doi:10.1016/S0092-8674(04)00044-3. 14744436

[2] Mariño-Ramírez L, Kann MG, Shoemaker B, Landsman D. 2005. Histone structure and nucleosome stability. Expert Rev Proteomics 2:719–729. doi:10.1586/14789450.2.5.719. 16209651 PMC1831843

[3] Dixon SE, Stilger KL, Elias E, Naguleswaran A, Sullivan WJ. 2010. A decade of epigenetic research in Toxoplasma gondii. Mol Biochem Parasitol 173:1–9. doi:10.1016/j.molbiopara.2010.05.001. 20470832 PMC2886187

[4] Luger K, Mäder AW, Richmond RK, Sargent DF, Richmond TJ. 1997. Crystal structure of the nucleosome core particle at 2.8 A resolution. Nature 389:251–260. doi:10.1038/38444. 9305837

[5] Zhou K, Gaullier G, Luger K. 2019. Nucleosome structure and dynamics are coming of age. Nat Struct Mol Biol 26:3–13. doi:10.1038/s41594-018-0166-x. 30532059 PMC7386248

[6] Harshman SW, Young NL, Parthun MR, Freitas MA. 2013. H1 histones: current perspectives and challenges. Nucleic Acids Res 41:9593–9609. doi:10.1093/nar/gkt700. 23945933 PMC3834806

[7] Fyodorov DV, Zhou B-R, Skoultchi AI, Bai Y. 2018. Emerging roles of linker histones in regulating chromatin structure and function. Nat Rev Mol Cell Biol 19:192–206. doi:10.1038/nrm.2017.94. 29018282 PMC5897046

[8] Aguirre AA, Longcore T, Barbieri M, Dabritz H, Hill D, Klein PN, Lepczyk C, Lilly EL, McLeod R, Milcarsky J, Murphy CE, Su C, VanWormer E, Yolken R, Sizemore GC. 2019. The One Health approach to toxoplasmosis: epidemiology, control, and prevention strategies. Ecohealth 16:378–390. doi:10.1007/s10393-019-01405-7. 30945159 PMC6682582

[9] Attias M, Teixeira DE, Benchimol M, Vommaro RC, Crepaldi PH, de Souza W. 2020. The life-cycle of Toxoplasma gondii reviewed using animations. Parasit Vectors 13:588. doi:10.1186/s13071-020-04445-z. 33228743 PMC7686686

[10] Radke JR, Behnke MS, Mackey AJ, Radke JB, Roos DS, White MW. 2005. The transcriptome of Toxoplasma gondii. BMC Biol 3:26. doi:10.1186/1741-7007-3-26. 16324218 PMC1325263

[11] Speer CA, Dubey JP. 2005. Ultrastructural differentiation of Toxoplasma gondii schizonts (types B to E) and gamonts in the intestines of cats fed bradyzoites. Int J Parasitol 35:193–206. doi:10.1016/j.ijpara.2004.11.005. 15710440

[12] Balaji S, Madan Babu M, Iyer LM, Aravind L. 2005. Discovery of the principal specific transcription factors of Apicomplexa and their implication for the evolution of the AP2-integrase DNA binding domains. Nucleic Acids Res 33:3994–4006. doi:10.1093/nar/gki709. 16040597 PMC1178005

[13] Radke JB, Worth D, Hong D, Huang S, Sullivan WJ, Wilson EH, White MW. 2018. Transcriptional repression by ApiAP2 factors is central to chronic toxoplasmosis. PLoS Pathog 14:e1007035. doi:10.1371/journal.ppat.1007035. 29718996 PMC5951591

[14] Sharma J, Rodriguez P, Roy P, Guiton PS. 2020. Transcriptional ups and downs: patterns of gene expression in the life cycle of Toxoplasma gondii. Microbes Infect 22:525–533. doi:10.1016/j.micinf.2020.09.001. 32931908

[15] Kim K. 2018. The epigenome, cell cycle, and development in Toxoplasma. Annu Rev Microbiol 72:479–499. doi:10.1146/annurev-micro-090817-062741. 29932347

[16] Hettmann C, Soldati D. 1999. Cloning and analysis of a Toxoplasma gondii histone acetyltransferase: a novel chromatin remodelling factor in Apicomplexan parasites. Nucleic Acids Res 27:4344–4352. doi:10.1093/nar/27.22.4344. 10536141 PMC148715

[17] Bhatti MM, Livingston M, Mullapudi N, Sullivan WJ. 2006. Pair of unusual GCN5 histone acetyltransferases and ADA2 homologues in the protozoan parasite Toxoplasma gondii. Eukaryot Cell 5:62–76. doi:10.1128/EC.5.1.62-76.2006. 16400169 PMC1360262

[18] Saksouk N, Bhatti MM, Kieffer S, Smith AT, Musset K, Garin J, Sullivan WJ, Cesbron-Delauw M-F, Hakimi M-A. 2005. Histone-modifying complexes regulate gene expression pertinent to the differentiation of the protozoan parasite Toxoplasma gondii. Mol Cell Biol 25:10301–10314. doi:10.1128/MCB.25.23.10301-10314.2005. 16287846 PMC1291236

[19] Dalmasso C, Echeverria PC, Zappia MP, Hellman U, Dubremetz JF, Angel SO. 2006. Toxoplasma gondii has two lineages of histones 2b (H2B) with different expression profiles. Mol Biochem Parasitol 148:103–107. doi:10.1016/j.molbiopara.2006.03.005. 16621068

[20] Brooks CF, Francia ME, Gissot M, Croken MM, Kim K, Striepen B. 2011. Toxoplasma gondii sequesters centromeres to a specific nuclear region throughout the cell cycle. Proc Natl Acad Sci USA 108:3767–3772. doi:10.1073/pnas.1006741108. 21321216 PMC3048097

[21] Talbert PB, Ahmad K, Almouzni G, Ausió J, Berger F, Bhalla PL, Bonner WM, Cande W, Chadwick BP, Chan SWL, Cross GAM, Cui L, Dimitrov SI, Doenecke D, Eirin-López JM, Gorovsky MA, Hake SB, Hamkalo BA, Holec S, Jacobsen SE, Kamieniarz K, Khochbin S, Ladurner AG, Landsman D, Latham JA, Loppin B, Malik HS, Marzluff WF, Pehrson JR, Postberg J, Schneider R, Singh MB, Smith M, Thompson E, Torres-Padilla M-E, Tremethick D, Turner BM, Waterborg J, Wollmann H, Yelagandula R, Zhu B, Henikoff S. 2012. A unified phylogeny-based nomenclature for histone variants. Epigenetics Chromatin 5:7. doi:10.1186/1756-8935-5-7. 22650316 PMC3380720

[22] Nardelli SC, Silmon de Monerri NC, Vanagas L, Wang X, Tampaki Z, Sullivan WJ, Angel SO, Kim K. 2022. Genome-wide localization of histone variants in Toxoplasma gondii implicates variant exchange in stage-specific gene expression. BMC Genomics 23:128. doi:10.1186/s12864-022-08338-6. 35164683 PMC8842566

[23] Dalmasso MC, Onyango DO, Naguleswaran A, Sullivan WJ, Angel SO. 2009. Toxoplasma H2A variants reveal novel insights into nucleosome composition and functions for this histone family. J Mol Biol 392:33–47. doi:10.1016/j.jmb.2009.07.017. 19607843 PMC2734877

[24] Hergeth SP, Schneider R. 2015. The H1 linker histones: multifunctional proteins beyond the nucleosomal core particle. EMBO Rep 16:1439–1453. doi:10.15252/embr.201540749. 26474902 PMC4641498

[25] Roque A, Iloro I, Ponte I, Arrondo LR, Suau P. 2005. DNA-induced secondary structure of the carboxyl-terminal domain of histone H1. J Biol Chem 280:32141–32147. doi:10.1074/jbc.M505636200. 16006555

[26] Kasinsky HE, Lewis JD, Dacks JB, Ausio J. 2001. Origin of H1 linker histones. FASEB J 15:34–42. doi:10.1096/fj.00-0237rev. 11149891

[27] Happel N, Doenecke D. 2009. Histone H1 and its isoforms: contribution to chromatin structure and function. Gene 431:1–12. doi:10.1016/j.gene.2008.11.003. 19059319

[28] Ponte I, Romero D, Yero D, Suau P, Roque A. 2017. Complex evolutionary history of the mammalian histone H1.1-H1.5 gene family. Mol Biol Evol 34:545–558. doi:10.1093/molbev/msw241. 28100789 PMC5400378

[29] Dalmasso MC, Joseph WSJ, Angel SO. 2011. Canonical and variant histones of protozoan parasites. Front Biosci (Landmark Ed) 16:2086–2105. doi:10.2741/3841. 21622164

[30] Andrés M, García-Gomis D, Ponte I, Suau P, Roque A. 2020. Histone H1 post-translational modifications: update and future perspectives. Int J Mol Sci 21:5941. doi:10.3390/ijms21165941. 32824860 PMC7460583

[31] Thorslund T, Ripplinger A, Hoffmann S, Wild T, Uckelmann M, Villumsen B, Narita T, Sixma TK, Choudhary C, Bekker-Jensen S, Mailand N. 2015. Histone H1 couples initiation and amplification of ubiquitin signalling after DNA damage. Nature 527:389–393. doi:10.1038/nature15401. 26503038

[32] Gutiyama LM, Chagas Da Cunha JP, Schenkman S. 2008. Histone H1 of Trypanosoma cruzi is concentrated in the nucleolus region and disperses upon phosphorylation during progression to mitosis. Eukaryot Cell 7:560–568. doi:10.1128/EC.00460-07. 18281601 PMC2292618

[33] Thiriet C, Hayes JJ. 2009. Linker histone phosphorylation regulates global timing of replication origin firing. J Biol Chem 284:2823–2829. doi:10.1074/jbc.M805617200. 19015270 PMC2631944

[34] Szerlong HJ, Herman JA, Krause CM, Deluca JG, Skoultchi A, Winger QA, Prenni JE, Hansen JC. 2015. Proteomic characterization of the nucleolar linker histone H1 interaction network. J Mol Biol 427:2056–2071. doi:10.1016/j.jmb.2015.01.001. 25584861 PMC4417401

[35] Kalashnikova AA, Rogge RA, Hansen JC. 2016. Linker histone H1 and protein-protein interactions. Biochim Biophys Acta 1859:455–461. doi:10.1016/j.bbagrm.2015.10.004. 26455956 PMC4775371

[36] Nardelli SC, Ting L-M, Kim K, Peacock C. 2015. Techniques to study epigenetic control and the epigenome in parasites, p 177–191. *In* Peacock C (ed), Parasite genomics protocols, 2nd ed. Springer, New York, NY. 10.1007/978-1-4939-1438-8_10 25388114

[37] Nishi M, Hu K, Murray JM, Roos DS. 2008. Organellar dynamics during the cell cycle of Toxoplasma gondii. J Cell Sci 121:1559–1568. doi:10.1242/jcs.021089. 18411248 PMC6810632

[38] de Monerri NCS, Yakubu RR, Chen AL, Bradley PJ, Nieves E, Weiss LM. 2015. The ubiquitin proteome of Toxoplasma gondii reveals roles for protein ubiquitination in cell-cycle transitions. Cell Host Microbe 18:621–633. doi:10.1016/j.chom.2015.10.014. 26567513 PMC4968887

[39] Treeck M, Sanders JL, Elias JE, Boothroyd JC. 2011. The phosphoproteomes of Plasmodium falciparum and Toxoplasma gondii reveal unusual adaptations within and beyond the parasites’ boundaries. Cell Host Microbe 10:410–419. doi:10.1016/j.chom.2011.09.004. 22018241 PMC3254672

[40] Watts E, Zhao Y, Dhara A, Eller B, Patwardhan A, Sinai AP. 2015. Novel approaches reveal that Toxoplasma gondii bradyzoites within tissue cysts are dynamic and replicating entities in vivo. mBio 6:e01155-15. doi:10.1128/mBio.01155-15. 26350965 PMC4600105

[41] Singh U, Brewer JL, Boothroyd JC. 2002. Genetic analysis of tachyzoite to bradyzoite differentiation mutants in Toxoplasma gondii reveals a hierarchy of gene induction. Mol Microbiol 44:721–733. doi:10.1046/j.1365-2958.2002.02903.x. 11994153

[42] Povelones ML, Gluenz E, Dembek M, Gull K, Rudenko G. 2012. Histone H1 plays a role in heterochromatin formation and VSG expression site silencing in Trypanosoma brucei. PLoS Pathog 8:e1003010. doi:10.1371/journal.ppat.1003010. 23133390 PMC3486875

[43] Kyte J, Doolittle RF. 1982. A simple method for displaying the hydropathic character of a protein. Mol Biol 157:105–132. doi:10.1016/0022-2836(82)90515-0. 7108955

[44] Shirai A, Matsuyama A, Yashiroda Y, Hashimoto A, Kawamura Y, Arai R, Komatsu Y, Horinouchi S, Yoshida M. 2008. Global analysis of gel mobility of proteins and its use in target identification. J Biol Chem 283:10745–10752. doi:10.1074/jbc.M709211200. 18292091

[45] Bogado SS, Dalmasso MC, Ganuza A, Kim K, Sullivan WJ, Angel SO, Vanagas L. 2014. Canonical histone H2Ba and H2A.X dimerize in an opposite genomic localization to H2A.Z/H2B.Z dimers in Toxoplasma gondii. Mol Biochem Parasitol 197:36–42. doi:10.1016/j.molbiopara.2014.09.009. 25286383 PMC4254167

[46] Ye X, Feng C, Gao T, Mu G, Zhu W, Yang Y. 2017. Linker histone in diseases. Int J Biol Sci 13:1008–1018. doi:10.7150/ijbs.19891. 28924382 PMC5599906

[47] Kalashnikova AA, Winkler DD, McBryant SJ, Henderson RK, Herman JA, Deluca JG, Luger K, Prenni JE, Hansen JC. 2013. Linker histone H1.0 interacts with an extensive network of proteins found in the nucleolus. Nucleic Acids Res 41:4026–4035. doi:10.1093/nar/gkt104. 23435226 PMC3627596

[48] Ni J, Liu L, Hess D, Rietdorf J, Sun F. 2006. Drosophila ribosomal proteins are associated with linker histone H1 and suppress gene transcription. Genes Dev 20:1959–1973. doi:10.1101/gad.390106. 16816001 PMC1522087

[49] Lu X, Wontakal SN, Emelyanov A, Morcillo P, Konev AY, Fyodorov Dv, Skoultchi AI. 2009. Linker histone H1 is essential for Drosophila development, the establishment of pericentric heterochromatin, and a normal polytene chromosome structure. Genes Dev 23:452–465. doi:10.1101/gad.1749309. 19196654 PMC2648648

[50] Grewal SIS, Jia S. 2007. Heterochromatin revisited. Nat Rev Genet 8:35–46. doi:10.1038/nrg2008. 17173056

[51] van Steensel B, Belmont AS. 2017. Lamina-associated domains: links with chromosome architecture, heterochromatin, and gene repression. Cell 169:780–791. doi:10.1016/j.cell.2017.04.022. 28525751 PMC5532494

[52] Fan Y, Nikitina T, Zhao J, Fleury TJ, Bhattacharyya R, Bouhassira EE, Stein A, Woodcock CL, Skoultchi AI. 2005. Histone H1 depletion in mammals alters global chromatin structure but causes specific changes in gene regulation. Cell 123:1199–1212. doi:10.1016/j.cell.2005.10.028. 16377562

[53] Shen X, Gorovsky MA. 1996. Linker histone H1 regulates specific gene expression but not global transcription in vivo. Cell 86:475–483. doi:10.1016/S0092-8674(00)80120-8. 8756729

[54] Gubbels MJ, Keroack CD, Dangoudoubiyam S, Worliczek HL, Paul AS, Bauwens C, Elsworth B, Engelberg K, Howe DK, Coppens I, Duraisingh MT. 2020. Fussing about fission: defining variety among mainstream and exotic apicomplexan cell division modes. Front Cell Infect Microbiol 10:269. doi:10.3389/fcimb.2020.00269. 32582569 PMC7289922

[55] Huang S, Holmes MJ, Radke JB, Hong D-P, Liu T-K, White MW, Sullivan WJ. 2017. Toxoplasma gondii AP2IX-4 regulates gene expression during bradyzoite development. mSphere 2:e00054-17. doi:10.1128/mSphere.00054-17. 28317026 PMC5352832

[56] Gubbels MJ, Wieffer M, Striepen B. 2004. Fluorescent protein tagging in Toxoplasma gondii: identification of a novel inner membrane complex component conserved among Apicomplexa. Mol Biochem Parasitol 137:99–110. doi:10.1016/j.molbiopara.2004.05.007. 15279956

[57] Goujon M, McWilliam H, Li W, Valentin F, Squizzato S, Paern J, Lopez R. 2010. A new bioinformatics analysis tools framework at EMBL-EBI. Nucleic Acids Res 8:695–699. doi:10.1093/nar/gkq313. PMC2896090 20439314

[58] Sievers F, Wilm A, Dineen D, Gibson TJ, Karplus K, Li W, Lopez R, McWilliam H, Remmert M, Söding J, Thompson JD, Higgins DG. 2011. Fast, scalable generation of high-quality protein multiple sequence alignments using Clustal Omega. Molecular Systems Biology 7:539. doi:10.1038/msb.2011.75. 21988835 PMC3261699

[59] Huynh MH, Carruthers VB. 2009. Tagging of endogenous genes in a Toxoplasma gondii strain lacking Ku80. Eukaryot Cell 8:530–539. doi:10.1128/EC.00358-08. 19218426 PMC2669203

[60] van den Hoff M, Moorman AFM, Lamers WH. 1992. Electroporation in “intracellular” buffer increases cell survival. Nucleic Acids Res 20:2902. doi:10.1093/nar/20.11.2902. 1614888 PMC336954

[61] Upadhya R, Kim K, Hogue-Angeletti R, Weiss LM. 2011. Improved techniques for endogenous epitope tagging and gene deletion in Toxoplasma gondii. J Microbiol Methods 85:103–113. doi:10.1016/j.mimet.2011.02.001. 21352857 PMC3073369

[62] Liffner B, Absalon S. 2021. Expansion microscopy reveals Plasmodium falciparum blood-stage parasites undergo anaphase with a chromatin bridge in the absence of mini-chromosome maintenance complex binding protein. Microorganisms 9:2306. doi:10.3390/microorganisms9112306. 34835432 PMC8620465

[63] Toro GC, Galanti N. 1988. H1 histone and histone variants in Trypanosoma cruzi. Exp Cell Res 174:16–24. doi:10.1016/0014-4827(88)90137-1. 3121371

[64] Obado SO, Field MC, Chait BT, Rout MP. 2016. High-efficiency isolation of nuclear envelope protein complexes from trypanosomes: methods and protocols. Methods Mol Biol 1411:315–324. 27147034 10.1007/978-1-4939-3530-7_3

[65] Villén J, Gygi SP. 2008. The SCX_IMAC enrichment approach for global phosphorylation. Nat Protoc 3:1630–1638. doi:10.1038/nprot.2008.150. 18833199 PMC2728452

[66] de Jesus TCL, Nunes VS, Lopes MDC, Martil DE, Iwai LK, Moretti NS, MacHado FC, de Lima-Stein ML, Thiemann OH, Elias MC, Janzen C, Schenkman S, da Cunha JPC. 2016. Chromatin proteomics reveals variable histone modifications during the life cycle of Trypanosoma cruzi. J Proteome Res 15:2039–2051. doi:10.1021/acs.jproteome.6b00208. 27108550

[67] Cox J, Mann M. 2008. MaxQuant enables high peptide identification rates, individualized p.p.b.-range mass accuracies and proteome-wide protein quantification. Nat Biotechnol 26:1367–1372. doi:10.1038/nbt.1511. 19029910

[68] Cox J, Hein MY, Luber CA, Paron I, Nagaraj N, Mann M. 2014. Accurate proteome-wide label-free quantification by delayed normalization and maximal peptide ratio extraction, termed MaxLFQ. Mol Cell Proteomics 13:2513–2526. doi:10.1074/mcp.M113.031591. 24942700 PMC4159666

[69] Karnovsky MJ. 1965. A formaldehyde-glutaraldehyde fixative of high osmolality for use in electron microscopy. J Cell Biol 27:137.

[70] Perez-Riverol Y, Bai J, Bandla C, García-Seisdedos D, Hewapathirana S, Kamatchinathan S, Kundu DJ, Prakash A, Frericks-Zipper A, Eisenacher M, Walzer M, Wang S, Brazma A, Vizcaíno JA. 2022. The PRIDE database resources in 2022: a hub for mass spectrometry-based proteomics evidences. Nucleic Acids Res 50:D543–D552. doi:10.1093/nar/gkab1038. 34723319 PMC8728295

