# Supplementary figures and images for: Previously Unidentified Histone H1-Like Protein Is Involved in Cell Division and Ribosome Biosynthesis in Toxoplasma gondii
Source: mSphere. 2022 Dec 5;7(6):e00403-22. doi: 10.1128/msphere.00403-22 (PMC9769792; doi:10.1128/msphere.00403-22)

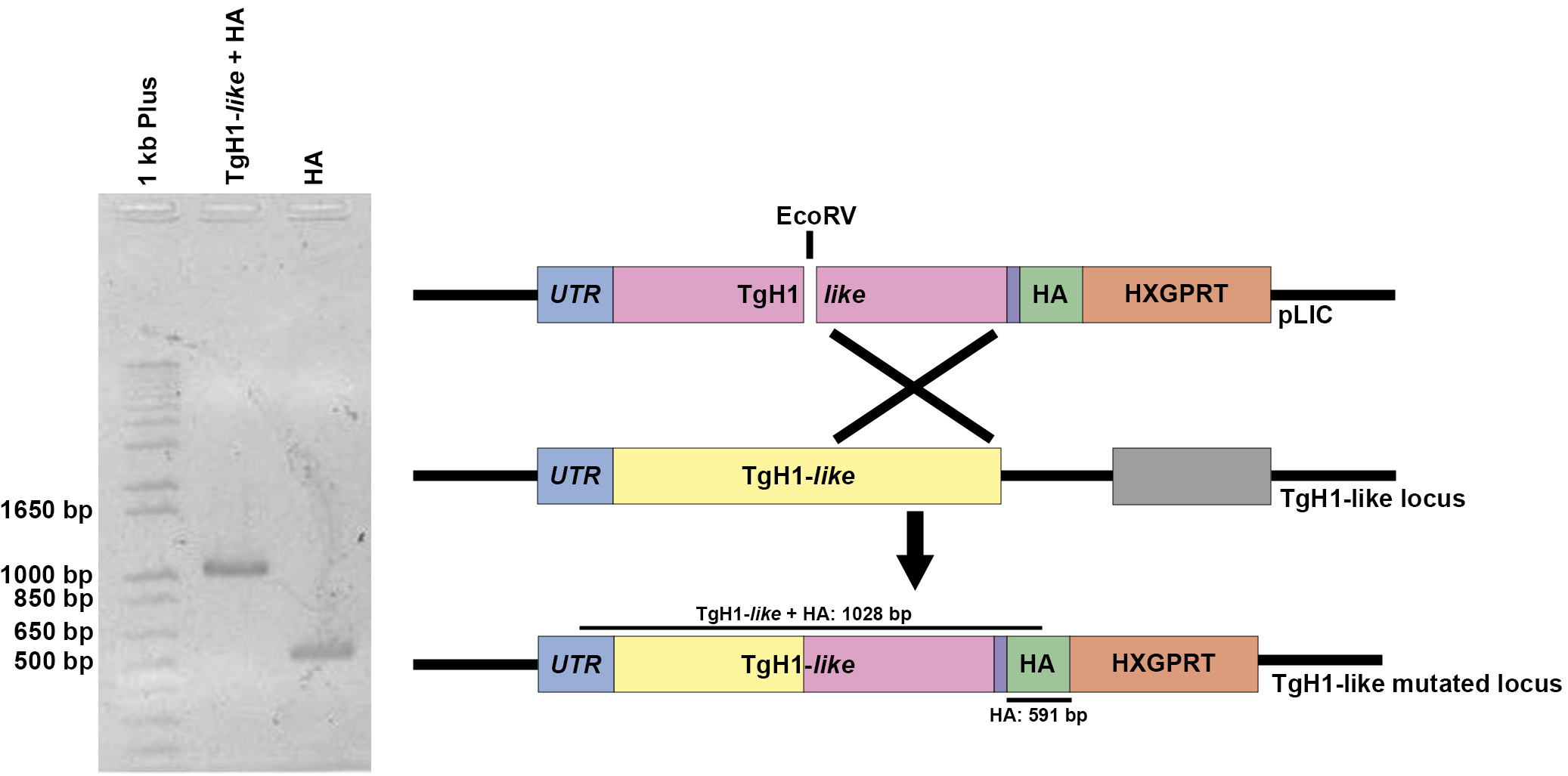

Supplement: FIG S1 [file msphere.00403-22-s0001.tif]

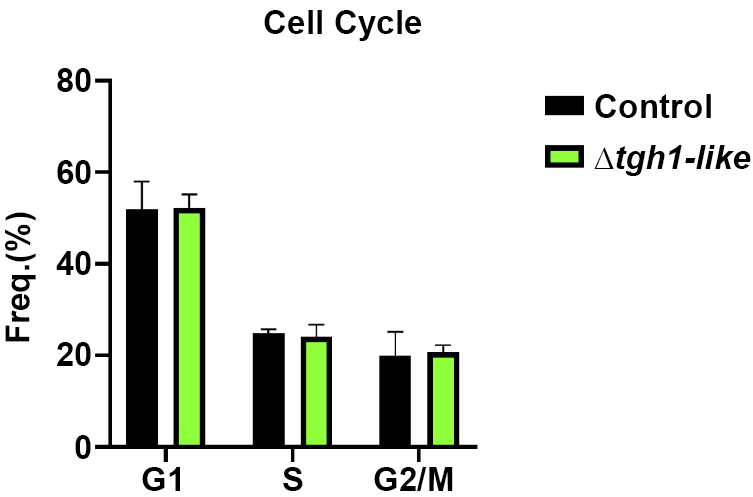

Supplement: FIG S2 [file msphere.00403-22-s0002.tif]
