# Supplementary material for: Previously Unidentified Histone H1-Like Protein Is Involved in Cell Division and Ribosome Biosynthesis in Toxoplasma gondii
Source: mSphere. 2022 Dec 5;7(6):e00403-22. doi: 10.1128/msphere.00403-22 (PMC9769792; doi:10.1128/msphere.00403-22)
Supplement: TABLE S2 [file msphere.00403-22-s0004.docx]

**Supplemental Material Table S2**. Primers used in this study

| **Primers** | **Sequence (5’-3’)** | **Used for** |
| --- | --- | --- |
| Tag TgH1-like - F | **TACTTCCAATCCAATTTAATGC**GTGTCGCACGAAGGAAATTC | To clone TGH1-like into pLIC-3XHA-HXGPRT (bold indicates LIC sequences). |
| Tag TgH1-like - R | **TCCTCCACTTCCAATTTTAGC**TTTCTTGCCCTTGCCCTT |  |
| HA-Int - R | GCATAATCGGGCACATCATAG | To confirm HA tag integration |
| HA - F | TACGACGTCCCGGACTACG |  |
| HA - R | AAGCTTATCGATACCGTCG |  |
| TgH1-like_S43A^HA^ - F | GCGAGCCGCAgcgGCGAAGCCTG | To insert site specific mutation into pLIC-TGH1-like-3XHA-HXGPRT (lowercase indicates mutation site). |
| TgH1-like_S43A^HA^ - R | TTGCACATGGTTTTCTTGGGTGCG |  |
| TgH1-like_S43A_K45R^HA^ F | gagaCCTGCAGCTGCCGCAGCT | To insert site specific mutation into pLIC-TGH1-like-3XHA-HXGPRT (lowercase indicates mutation site). |
| TgH1-like_S43A_K45R^HA^ R | gccgcTGCGGCTCGCTTGCACATG |  |
| TgH1-like 5’ UTR - F | ACGGCGATGTCGGAGG | To amplify the 5’ UTR of *TGH1*-like for knockout (lowercase indicates *HXGPRT* sequence and uppercase indicates TGH1-like sequence). |
| TgH1-like 5’ UTR - R | ccgcgggcgggtttgaatgcaaggtttcgtgctgTGTGGAGGACTTGGAGGG |  |
| TgH1-like 3’ UTR - F | ccgcgggcgggtttgaatgcaaggtttcgtgctgTGTGGAGGACTTGGAGGG | To amplify the 3’ UTR of *TGH1*-like for knockout (lowercase indicates *HXGPRT* sequence and uppercase indicates TGH1-like sequence). |
| TgH1-like 3’ UTR - R | TCACTGAAAAGGGGGCAC |  |
| *HXGPRT* - F | CCAAACCCATTGAAGACTAC | To confirm the presence of the selectable marker *HXGPRT* and correct integration. |
| *HXGPRT* - R | GAAAAGAGAGGAAAACGAGAGA |  |
| *HXGPRT*-Int | CCCGAGAAAAAGAAACGAACAGA |  |
